# Frequent ploidy changes in Salicaceae indicates widespread sharing of the salicoid whole genome duplication by the relatives of *Populus* L. and *Salix* L.

**DOI:** 10.1186/s12870-021-03313-x

**Published:** 2021-11-13

**Authors:** Zhong-Shuai Zhang, Qing-Yin Zeng, Yan-Jing Liu

**Affiliations:** State Key Laboratory of Tree Genetics and Breeding, Chinese Academy of Forestry, 100091 Beijing, China

**Keywords:** Salicaceae, Chromosome number, DNA content, Phylogeny, Whole genome duplication

## Abstract

**Backgrounds:**

*Populus* and *Salix* belong to Salicaceae and are used as models to investigate woody plant physiology. The variation of karyotype and nuclear DNA content can partly reflect the evolutionary history of the whole genome, and can provide critical information for understanding, predicting, and potentially ameliorating the woody plant traits. Therefore, it is essential to study the chromosome number (CN) and genome size in detail to provide information for revealing the evolutionary process of Salicaceae.

**Results:**

In this study, we report the somatic CNs of seventeen species from eight genera in Salicaceae. Of these, CNs for twelve species and for five genera are reported for the first time. Among the three subfamilies of Salicaceae, the available data indicate CN in Samydoideae is *n* = 21, 22, 42. The only two genera, *Dianyuea* and *Scyphostegia*, in Scyphostegioideae respectively have *n* = 9 and 18. In Salicoideae, *Populus*, *Salix* and five genera closely related to them (*Bennettiodendron*, *Idesia*, *Carrierea*, *Poliothyrsis*, *Itoa*) are based on relatively high CNs from *n* = 19, 20, 21, 22 to *n* = 95 in *Salix*. However, the other genera of Salicoideae are mainly based on relatively low CNs of *n* = 9, 10, 11. The genome sizes of 35 taxa belonging to 14 genera of Salicaceae were estimated. Of these, the genome sizes of 12 genera and all taxa except *Populus euphratica* are first reported. Except for *Dianyuea*, *Idesia* and *Bennettiodendron*, all examined species have relatively small genome sizes of less than 1 pg, although polyploidization exists.

**Conclusions:**

The variation of CN and genome size across Salicaceae indicates frequent ploidy changes and a widespread sharing of the salicoid whole genome duplication (WGD) by the relatives of *Populus* and *Salix*. The shrinkage of genome size after WGD indicates massive loss of genomic components. The phylogenetic asymmetry in clade of *Populus*, *Salix*, and their close relatives suggests that there is a lag-time for the subsequent radiations after the salicoid WGD event. Our results provide useful data for studying the evolutionary events of Salicaceae.

**Supplementary Information:**

The online version contains supplementary material available at 10.1186/s12870-021-03313-x.

## Background

*Populus* L. (poplars) and *Salix* L. (willows) include important woody trees with enormous ecological and economic value. The two genera have many valuable characteristics, such as fast growth, easy propagation, hybridization, pleasing appearance, and widespread distribution. These features make them important fiber resources, fuelwood, and candidates for ecological management projects, for example the rehabilitation of degraded land and the mitigation of climate change [1]. They also have important research value in the fields of wood formation, long-term perennial growth and seasonality. They are model systems of woody plant genetics, genomics, and biology [2].

The evolutionary history of poplars and willows is the basic biological roadmap to guide the disclosure of woody plant traits. A reliable phylogenetic relationship among poplar, willow and their relatives is essential, but it has just surfaced. The now expended Salicaceae includes three subfamilies, Samydoideae, Scyphostegioideae and Salicoideae [3]. Phylogenetic analysis based on molecular or morphological data resolved *Populus* and *Salix* as sister genera, and they are deeply nested in Salicoideae [3–6]. And the immediate sister groups to the clade containing poplars and willows are resolved as some genera that are apetalous, unisexual, and mainly dioecious, namely *Poliothyrsis* Oliv., *Itoa* Hemsl., *Carrierea* Franch., *Idesia* Maxim., *Bennettiodendron* Merr., *Olmediella* Baill., and *Macrohasseltia* L.O. Williams [4, 7, 8]. However, under different analysis methods and taxon sampling densities, the sister taxon with *Populus* and *Salix* remains controversial [3, 6–9].

Polyploidy or whole-genome duplication (WGD) is an important source for adaptation, speciation and evolution in plants [10]. Studies based on chromosome numbers suggested that ca. 30 % to perhaps 70 % of angiosperm are of polyploid origin [11–13]. Recent genome- and transcriptome-based analyses revealed that angiosperm contains at least one paleopolyploid event and lineage-specific polyploidy events are widespread [14–16]. Changes in gene expression and epigenetics after polyploidization can affect the morphology and physiology of polyploidies which in turn has the potential to affect the bio-environment and interspecies interactions [17–20]. Several ancient genome-doubling events have been proved to be closely related to evolution radiation and diversification in many angiosperm lineages such as Poaceae, Solanaceae, Fabaceae, and Brassicaceae [14, 21]. In Malpighiales, which Salicaceae belongs to, Cai et al. [22] identified 22 ancient WGD events which clustered around the Eocene–Paleocene transition, during which time the planet was warmer and wetter than any period in the Cenozoic. And these WGDs are usually associated with the most diverse clades in Malpighiales, for example, the clusioids, ochnoids, euphorbioids, phyllanthoids, violets, and passion flowers. The salicoid WGD event is inferred to predate the common ancestor of *Populus* and *Salix*. However, it remains unclear whether this WGD event is shared by other taxa of Salicaceae [22].

Diversification and speciation of plants are often accompanied by variations in the chromosome number and structure, together with the amount of nuclear DNA. Nuclear genome size, i.e. the DNA content of the unreplicated nucleus, 2 C [23], is an important genomic parameter that exhibits pronounced variation among angiosperm with a minimum of 1 C = 0.07 pg in *Genlisea aurea* [24] and a known maximum of 1 C = 152.23 pg in *Paris japonica* [25]. There is an increased interest in its evolutionary potential in the last decade [26–31]. For example, a study using 219 geophytes indicated a positive correlation between stomatal and genome size, and increased genome size was associated with earliness of flowering and tendency to grow in humid conditions [32]. In *Veronica*, life history is significantly correlated with 1 C-value, and significant genome downsizing accompanied by increased diversification rates exist in the polyploid Southern Hemisphere subgenus *Pseudoveronica* and two Northern Hemisphere subgenera [33]. Thus, assessments of the karyotype and nuclear DNA content are traditional and useful methods to explore genetic relationships and polyploid events [34, 35]. In *Populus*, the chromosome number of 24 species from five sections are known [36, 37]. They all have the basic chromosome number (BCN) *x* = 19, and the majority individuals are diploid, except in the north American aspen *P. tremuloides* Michx., triploids are widespread in unglaciated, drought-prone regions [38]. In *Salix*, the situation is much more complicated. Although most species are based on *x* = 19, BCN of *x* = 22 also appears in some species. In some extreme examples, different BCNs may present in the same species [39–41]. However, the chromosome number (CN) data of other Salicaceae genera are very insufficient (Supplementary Table S1). The pantropical Samydoideae includes 13 genera and ca. 235 species. Only one genus (7.7 %) and five species (2.1 %) have CN reports. In Salicoideae, which includes 40 genera and more than 960 species, there is few attention on cytology of the taxa except *Populus* and *Salix*. Only 9 genera (22.5 %), and 28 taxa (excluding the 201 taxa of *Populus* or *Salix*) have CN reports. The close relatives of *Populus* and *Salix* include *Itoa*, *Poliothyrsis*, *Carrierea*, *Idesia*, *Bennettiodendron*, *Macrohasseltia* and *Olmediella* [5]. Of these seven genera, there is only an uncertain CN report for *Idesia polycarpa* Maxim. [42]. Genome size, defined as the DNA mass in picograms within an un-replicated gametic nucleus, is a basic and important metric for comparing plant genomes and can provide insight into the evolutionary history of plants [23]. Kew Plant DNA C-value database [43] is a widely used resource that contains many past and current estimates of genome size. The database currently contains C-value data for 12,273 species comprising 10,770 angiosperms, 421 gymnosperms, 303 pteridophytes, 334 bryophytes, and 445 algae [43]. In Salicaceae, only three genera (*Populus*, *Salix*, and *Casearia* Jacq.) and 24 species have a genome size report ([43]; Supplementary Table S2). The lack of recognition of chromosome number and DNA content in Salicaceae hinders our understanding of the role of polyploidization in the evolution of Salicaceae.

In this article, we intend to study the chromosome number and genome size of Salicaceae especially the close relatives of *Populus* and *Salix*. We want to give a more precise process by which the chromosome number changes under the phylogenetic framework; and we intend to uncover the phylogenetic placements of the salicoid WGD.

## Results

### Somatic Karyotypes in Salicaceae

Chromosome number (CN) has been the most influential and ease-obtain data for detecting major genomic events, such as whole genome duplication (WGD). To reveal the evolutionary history of *Populus* genomes, we explored the dynamic changes of CN in Salicaceae. The phylogeny of Salicaceae and the likely sister family Lacistemataceae is presented following previous studies [3, 44–47]. We collected available karyotypes of Salicaceae and Lacistemataceae species from online database, Chromosome Counts Database (Supplementary Table S1). In addition, we detected the chromosome numbers of seventeen species from eight genera by cytological analysis (Table 1). These eight genera include *Populus*, *Itoa*, *Poliothyrsis*, *Carrierea*, *Idesia*, *Bennettiodendron*, *Dianyuea*, and *Casearia*. Among the 17 species, ten were selected from three sections (sect. *Populus*, sect. *Tacamahaca* and sect. *Leucoides*) of *Populus*. Five species from monotypic or oligotypic genera *Itoa*, *Poliothyrsis*, *Carrierea*, *Idesia*, *Bennettiodendron*, which are considered closely related to *Populus* and *Salix*, were sampled. The last two species were from the monotypic genus *Dianyuea* of Scyphostegioideae (includes two monotypic genera) and the big pantropical genus *Casearia* of Samydoideae (includes 13 genera and 235 species), respectively (Table 1). Of these taxa, the CN for five genera (*Itoa*, *Poliothyrsis*, *Carrierea*, *Bennettiodendron*, and *Dianyuea*) are reported for the first time.

**Table 1:** Somatic karyotypes of studied taxa

Species	Collection data [herbarium with voucher specimen]	Somatic karyotype
*Casearia velutina* Bl.^a^	Menghai County, Yunnan Province, China, Sep. 2020, 1675 m, [CAF *Zhang Zhongshuai* *20200926-1*]	2*n* = 44
*Dianyuea turbinata* (H.J. Dong & H. Peng) C. Shang, S. Liao & Z.X. Zhang^b^	Yingjiang County, Yunnan Province, China, July 2020, 1412 m, [CAF *Zhang Zhongshuai* *20200701-1*]	2*n* = 38
*Bennettiodendron leprosipes* (Clos) Merr.^b^	Malipo County, Yunnan Province, China, June 2021, 1200 m, [CAF *Xiao Bo 20210620-1*]	2*n* = 42
*Idesia polycarpa* Maxim.^b^	Baoxing, Sichuan Province, China, July 2020, 1364 m, [CAF *Zhang Zhongshuai 20200718-12*]	2*n* = 42
	Baoxing, Sichuan Province, China, July 2020, 1364 m, [CAF *Zhang Zhongshuai 20200718-13*]	2*n* = 42
	Wenxian, Gansu Province, China, July 2020, 765 m, [CAF *Zhang Zhongshuai 20200725-4*]	2*n* = 42
*Carrierea calycina* Franch.^b^	Baoxing County, Sichuan Province, China, July 2020, 1364 m, [CAF *Zhang Zhongshuai 20200718-14*]	2*n* = 40
*Poliothyrsis sinensis* Oliv.^b^	Shennongjia County, Hubei Province, China, May 2021, 1204 m, [CAF *Zhang Zhongshuai 20210504-1*]	2*n* = 40
*Itoa orientalis* Hemsl.^b^	Jingxi City, Guangxi Province, China, August 2020, 800 m, [CAF *Jiang Rihong 20200828-1*]	2*n* = 40
*Populus adenopoda* Maxim.	Wenxian County, Gansu Province, China, July 2020, 765 m, [CAF *Zhang Zhongshuai 20200725-1*]	2*n* = 38
*Populus cathayana* Rehder	Meixian County, Shannxi Province, China, July 2020, 2182 m, [CAF *Zhang Zhongshuai 20200725-1*]	2*n* = 38
*Populus ciliata* Wall. ex Royle	Luozha County, Xizang Province, China, June 2020, 2844 m, [CAF *Zhang Zhongshuai 20200619-22*]	2*n* = 38
*Populus davidiana* Dode	Wenxian, Gansu Province, China, July 2020, 715 m, [CAF *Zhang Zhongshuai 20200725-13*]	2*n* = 38
*Populus glauca* Haines^a^	Dingjie County, Xizang Province, China, June 2020, 2867 m, [CAF *Zhu Xinxin 20200615-1*]	2*n* = 38
*Populus pamirica* Kom.^a^	Aketao County, Xinjiang Province, China, May 2021, 2737 m, [CAF *Zhang Zhongshuai 20210518-10*]	2*n* = 38
*Populus szechuanica* Schneid.^a^	Wenchuan County, Sichuan Province, China, July 2020, 2223 m, [CAF *Zhang Zhongshuai 20200717-47*]	2*n* = 38
*Populus wilsonii* Schneid.^a^	Meixian, Shannxi Province, China, August 2020, 2491 m, [CAF *Zhang Zhongshuai 20200801-1*]	2*n* = 38
*Populus yatungensis* (Z. Wang & P.Y. Fu) C. Wang & S.L. Tung^a^	Yadong County, Xizang Province, China, June 2020, 3430 m, [CAF *Zhang Zhongshuai 20200616-3*]	2*n* = 38
*Populus yunnanensis* Dode^a^	Tengchong Shi, Yunnan Province, China, April 2021, 2385 m, [CAF *Zhang Zhongshuai 20210417-10*]	2*n* = 38

^a^ The chromosome numbers of these species are reported for the first time

^b^ The chromosome numbers of the genera represented by these species are reported or confirmed for the first time

**Fig. 1:**
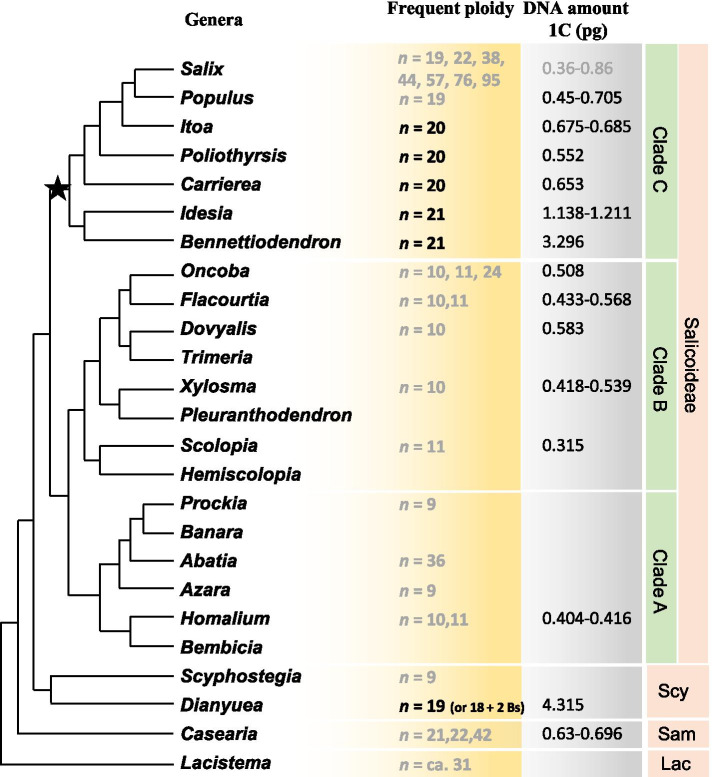
A summary phylogeny based on published trees of Salicaceae [3, 46]. The inferred chromosome numbers superimposed onto the phylogenetic tree are predominant counts for each genus based on available data. The chromosome numbers and mean 1 C DNA amounts assigned based on cited publications or database are in grey. Hypothesized placement of the salicoid WGD event is indicated with star. Scy, Scyphostegioideae. Sam, Samydoideae. Lac, Lacistemataceae

**Fig. 2:**
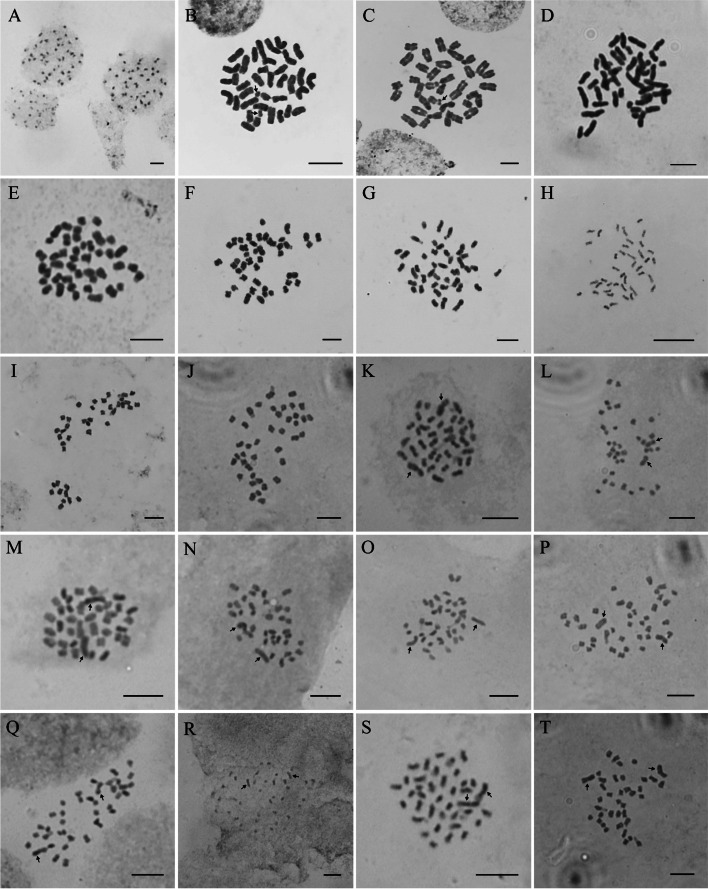
Somatic chromosomes of *Populus* and its relatives. **A** *Casearia velutina* (2*n* = 44). **B**-**C** *Dianyuea turbinata* (2*n* = 38). **D** *Bennettiodendron leprosipes* (2*n* = 42). **E**-**G** *Idesia polycarpa* (2*n* = 42). **H** *Itoa orientalis* (2*n* = 40). **I** *Carrierea calycina* (2*n* = 40). **J** *Poliothyrsis sinensis* (2*n* = 40). **K** *Populus adenopoda* (2*n* = 38). **L** *P. cathayana* (2*n* = 38). **M** *P. ciliata* (2*n* = 38). **N** *P. davidiana* (2*n* = 38). O *P. glauca* (2*n* = 38). **P** *P. pamirica* (2*n* = 38). **Q** *P. szechuanica* (2*n* = 38). **R** *P. wilsonii* (2*n* = 38). **S** *P. yatungensis* (2*n* = 38). **T** *P. yunnanensis* (2*n* = 38). Arrows in **B**-**C** indicate the possible extra-chromosomes, and in **K**-**T** indicate the two large chromosomes of poplars

Salicaceae includes three subfamilies, Salicoideae, Scyphostegioideae, and Samydoideae (Fig. 1). Samydoideae has thirteen genera and ca. 235 species [3, 48]. Five species of *Casearia*, a big pantropical genus in Samydoideae with ca. 180 species, were reported to have 42, 44, or 84 chromosomes in somatic cells (Supplementary Table S1). We also confirm that *C. velutina* Blume has CN of 2*n* = 44 (Table 1). The Scyphostegioideae has two monotypic genera, *Scyphostegia* Stapf and *Dianyuea*. The somatic karyotype of *Scyphostegia* is 2*n* = 18, and CN of *Dianyuea* is 2*n* = 38 or 2*n* = 36 +2B. The chromosomes of *D. turbinata* (H.J.Dong & H.Peng) C.Shang, S.Liao & Z.X.Zhang are relatively larger than the other species studied (Fig. 2). In Salicoideae, clade A includes six genera (*Bembicia* Oliv., *Homalium* Jacq., *Azara* Ruiz & Pav., *Abatia* Ruiz & Pav., *Banara* Aubl., and *Prockia* P. Browne ex L.), clade B includes eight genera (*Hemiscolopia* D. F. van Slooten, *Scolopia* Schreb., *Pleuranthodendron* L.O. Williams, *Xylosma* G. Forst., *Trimeria* Harv., *Dovyalis* E. Mey. ex Arn., *Flacourtia* Comm. ex L’Hér., and *Oncoba* Forssk.), and clade C includes seven genera (*Bennettiodendron*, *Idesia*, *Poliothyrsus*, *Itoa*, *Carrierea*, *Populus* and *Salix*). In clade A, species from *Azara* and *Prockia* are identified with 2*n* = 18, species of *Homalium* are based on *n* = 10 or 11, while a species of *Abatia* has *n* = ca. 36. In clade B, *Scolopia*, *Xylosma*, *Dovyalis*, *Flacourtia*, and *Oncoba* are based on *n* = 10 or 11, except for the CN of *O. dentata* Oliv. (2*n* = 48), which seems to be a tetraploid (4*x* = 48) [49]. In clade C, *Bennettiodendron* and *Idesia* share the CN of 2*n* = 42, which is based on *n* = 21. *Poliothyrsus*, *Itoa* and *Carrierea* share a CN of 2*n* = 40, which could assume to be derived from the basic chromosome number of *n* = 21 of *Bennettiodendron* and *Idesia* by chromosome fusions [50]. Combining our results with online data, the CN of *Populus* and *Salix* are *n* = 19, and *n* = 19, 22, 38, 44, 57, 76, 95 respectively. All the *Populus* species have two obviously long chromosomes as indicated by arrows in Fig. 2, which are not present in poplar relatives studied in this study (Fig. 2). Finally, as an outgroup, the *Lacistema aggregatum* (P.J. Bergius) Rusby from Lacistemataceae has a chromosome count of 2*n* = ca. 62.

### Salicaceae DNA C-values

**Fig. 3:**
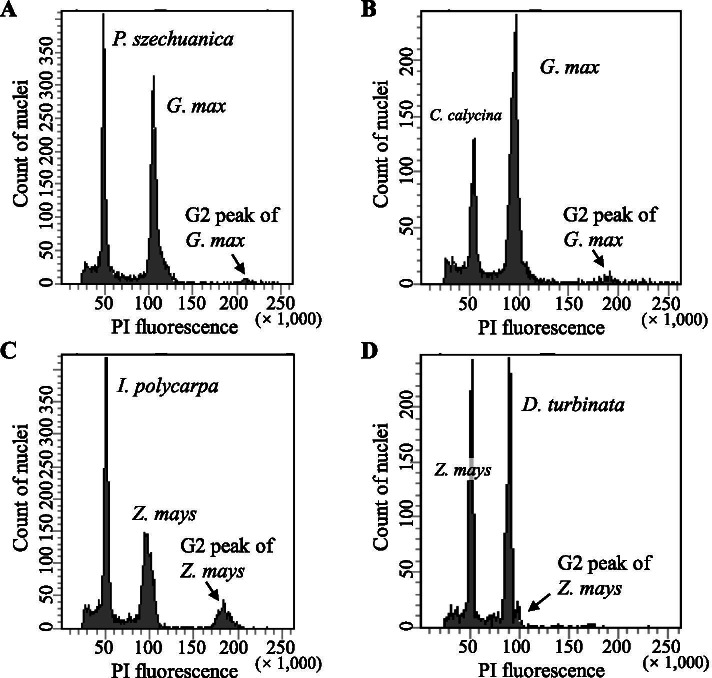
Estimation of nuclear DNA amount (genome size). The histograms of PI fluorescence were obtained by flow cytometric analysis. *Glycine max* Williams 82 served as internal standard for *Populus szechuanica* (**A**) and *Carrierea calycina* (**B**), while *Zea mays* L. B73 served as internal standard for *Idesia polycarpa* (**C**) and *Dianyuea turbinate* (**D**). Small peaks at about twice the DNA content of the marker G1 peak represent the cells in G2 phase

Besides chromosome number, we observed representative signatures of chromosome size in different Salicaceae species (Fig. 1; Table 2). By searching the Plant DNA C-values database of Kew, we found only species from three genera of Salicaceae have DNA C-values estimates [43]. The 1 C DNA amount of *Casearia bourdillonii* Mukh. (Samydoideae) is 0.63 pg. The 1 C DNA amount varies from 0.45 pg in *P. tremula* L. to 0.55 pg in *P. tremuloides* in *Populus*, and it varies from 0.36 pg in *S. amygdaloides* Andersson (2*n* = 38) to 0.86 pg in *S.* ×*fragilis* L. (2*n* = 38, 76, 114) in *Salix* ([43]; Supplementary Table S2). In this study, we estimate the DNA C-values of 35 taxa from 14 genera of Salicaceae by flow cytometric analysis, as illustrated in Fig. 3. In Samydoideae, the 1 C DNA amount of *Casearia graveolens* Dalz. is 0.696 pg, which is similar to that of *C. bourdillonii*. In Scyphostegioideae, the 1 C DNA amount of *Dianyuea turbinata* is 4.315 pg, which is the biggest in Salicaceae. In Salicoideae clade A, two resources of *Homalium ceylanicum* (Gardner) Benth. have 1 C DNA amount of 0.416 pg in resource C17057 and 0.404 pg in 00GN0039, respectively. However, there is no statistically significance difference between the two resources (*p* = 0.136, *t*-test). In Salicoideae clade B, 1 C DNA contents of five genera range from 0.315 pg in *Scolopia chinensis* (Lour.) Clos to 0.568 pg in *Flacourtia indica* (Burm. f.) Merr. And they are different from each other (*p* < 0.002, *t*-test) except for *Xylosma* and *Oncoba* (*p* = 0.498, *t*-test). In Salicoideae clade C, *Bennettiodendron* (*n* = 21, 1 C DNA amount = 3.296 pg) and *Idesia* (*n* = 21, 1 C DNA amount = 1.138-1.211 pg) have relatively large 1 C DNA amounts with almost six and two times of the average (about 0.530 pg) of the other five genera, respectively. The rest five genera have similar 1 C DNA amounts, varying from 0.552 pg in *Poliothyrsis* to 0.685 pg in *Itoa*. The 1 C DNA amount of *Populus* varies from 0.45 pg in *P. tremula* to 0.577 pg in *P. euphratica* Olivier, except 0.705 pg in an individual of *P. suaveolens* Fisch. which probably represent a triploid. In *Salix*, it varies from 0.36 to 0.86 pg, which reflect the frequent polyploidy in the genus.

**Table 2:** The 1 C nuclear DNA amounts (the amount of DNA contained within un-replicated haploid chromosome set) of studied taxa

Species	Collection data [herbarium with voucher specimen]	Individuals examined (pg, test replicates)	DNA amount 1 C (pg, means ± SD)
*Casearia graveolens* Dalz. ^a^	Cultivated in XTBG, resource number: 0020010492 (CAF)	A (0.703, 0.682, 0.704)	0.696 ± 0.012
*Dianyuea turbinata* (H.J. Dong & H. Peng) C. Shang, S. Liao & Z.X. Zhang^b^	Yingjiang County, Yunnan Province, China, July 2020, 1412 m, [CAF *Zhang Zhongshuai* *20200701-1*]	A (4.400, 4.275) B (4.408, 4.243) C (4.369, 4.194)	4.315 ± 0.090
*Homalium ceylanicum* (Gardner) Benth.^b^	Cultivated in XTBG, resource number: C17057 (CAF)	A (0.416, 0.420) B (0.419, 0.410)	0.416 ± 0.005
	Cultivated in XTBG, resource number: 00GN0039 (CAF)	A (0.398, 0.419, 0.396)	0.404 ± 0.013
*Scolopia chinensis* (Lour.) Clos^b^	Cultivated in XTBG, resource number: 0020023293 (CAF)	A (0.310, 0.320, 0.315)	0.315 ± 0.005
*Xylosma congesta* (Lour.) Merr.^b^	Cultivated in KIB (CAF)	A (0.422, 0.413)	0.418 ± 0.006
*Xylosma longifolia* Clos^b^	Cultivated in XTBG, resource number: 0020090847 (CAF)	A (0.508, 0.537, 0.511) B (0.547, 0.54, 0.542)	0.531 ± 0.017
	Cultivated in XTBG, resource number: 0020030017 (CAF)	A (0.527, 0.538, 0.524)	0.530 ± 0.007
	Cultivated in XTBG, resource number: 0020000109 (CAF)	A (0.537, 0.545, 0.535)	0.539 ± 0.005
*Dovyalis caffra* (Hook. f. & Harv.) Warb.^b^	Cultivated in XTBG, resource number: 1,520,080,001 (CAF)	A (0.585, 0.574) B (0.569, 0.595, 0.571) C (0.573, 0.592) D (0.579, 0.602, 0.586)	0.583 ± 0.011
*Flacourtia indica* (Burm. f.) Merr.^b^	Cultivated in XTBG, resource number: 0020081213 (CAF)	A (0.57, 0.583, 0.548) B (0.573, 0.581, 0.555)	0.568 ± 0.014
*Flacourtia inermis* Roxb.^b^	Cultivated in XTBG, resource number: 3,720,040,116 (CAF)	A (0.456, 0.49, 0.454) B (0.470, 0.492, 0.458)	0.470 ± 0.017
*Flacourtia rukam* Zoll. & Moritzi^b^	Cultivated in XTBG, resource number: 0020023270 (CAF)	A (0.440, 0.442, 0.436) B (0.438, 0.453, 0.437) C (0.460, 0.448, 0.434) D (0.436, 0.458, 0.433)	0.433 ± 0.009
	Cultivated in XTBG, resource number: 0020000597 (CAF)	A (0.454, 0.449, 0.438) B (0.440, 0.463, 0.438) C (0.437, 0.445, 0.432) D (0.455, 0.434, 0.436)	0.443 ± 0.01
*Oncoba echinata* Oliver.^b^	Cultivated in XTBG, resource number: 0320130007 (CAF)	A (0.514, 0.512, 0.508) B (0.512, 0.521, 0.499) C (0.505, 0.500, 0.500)	0.508 ± 0.008
*Bennettiodendron leprosipes* (Clos) Merr.^b^	Cultivated in XTBG, resource number: 0020020036 (CAF)	A (3.298, 3.243, 3.277) B (3.321, 3.296, 3.339)	3.296 ± 0.034
*Idesia polycarpa* Maxim.^b^	Baoxing, Sichuan Province, China, July 2020, 1364 m, [CAF *Zhang Zhongshuai 20200718-13*]	A (1.221, 1.249) B (1.142, 1.232)	1.211 ± 0.047
	Wenxian, Gansu Province, China, July 2020, 765 m, [CAF *Zhang Zhongshuai 20200725-4*]	A (1.066, 1.210)	1.138 ± 0.102
*Carrierea calycina* Franch.^b^	Baoxing County, Sichuan Province, China, July 2020, 1364 m, [CAF *Zhang Zhongshuai 20200718-14*]	A (0.662, 0.650) B (0.658, 0.640)	0.653 ± 0.01
*Poliothyrsis sinensis Oliv.* ^b^	Cultivated in KIB (CAF)	A (0.575, 0.559, 0.536) B (0.559, 0.535) C (0.558, 0.542) D (0.558, 0.54) E (0.559, 0.548)	0.552 ± 0.012
*Itoa orientalis* Hemsl.^b^	Cultivated in XTBG, resource number: 0020140861 (CAF)	A (0.678, 0.689, 0.672) B (0.668, 0.673, 0.669)	0.675 ± 0.008
	Jingxi City, Guangxi Province, China, August 2020, 800 m, [CAF *Jiang Rihong 20200828-1*]	A (0.678, 0.677) B (0.692, 0.702) C (0.678, 0.686, 0.680)	0.685 ± 0.009
*Populus adenopoda* Maxim.^a^	Wenxian County, Gansu Province, China, July 2020, 765 m, [CAF *Zhang Zhongshuai 20200725-1*]	A (0.471, 0.474) B (0.461, 0.467) C (0.467, 0.476)	0.469 ± 0.005
*Populus cathayana* Rehder^a^	Diebu County, Gansu Province, China, July 2020, 2209 m, [CAF *Zhang Zhongshuai 20200727-7*]	A (0.493, 0.487)	0.49 ± 0.004
	Wenchuan County, Sichuan Province, China, July 2020, 1926 m, [CAF *Zhang Zhongshuai 20200717-43*]	A (0.491)	0.491
	Kangding, Sichuan Province, China, July 2020, 3116 m, [CAF *Zhang Zhongshuai 20200721-13*]	A (0.500, 0.505)	0.503 ± 0.004
	Meixian County, Shannxi Province, China, July 2020, 2182 m, [CAF *Zhang Zhongshuai 20200725-1*]	A (0.494, 0.500)	0.497 ± 0.004
	Luding County, Sichuan Province, China, July 2020, 1512 m, [CAF *Zhang Zhongshuai 20200720-1*]	A (0.509, 0.505, 0.507)	0.507 ± 0.002
*Populus ciliata* Wall. ex Royle^a^	Jilong County, Xizang Province, China, June 2020, 1841 m, [CAF *Zhang Zhongshuai 20200610-10*]	A (0.533, 0.534) B (0.526, 0.534)	0.532 ± 0.004
	Luozha County, Xizang Province, China, June 2020, 2844 m, [CAF *Zhang Zhongshuai 20200619-22*]	A (0.513, 0.516, 0.520)	0.516 ± 0.004
*Populus davidiana* Dode^a^	Wenxian, Gansu Province, China, July 2020, 715 m, [CAF *Zhang Zhongshuai 20200725-13*]	A (0.461, 0.470) B (0.470, 0.475) C (0.455, 0.473)	0.467 ± 0.008
*Populus euphratica* Olivier	Zhongwei, Ningxia Province, China, July 2020, 1400 m, [CAF *Zhang Zhongshuai*]	A (0.580, 0.568) B (0.580, 0.584) C (0.571, 0.581)	0.577 ± 0.006
*Populus glauca* Haines^a^	Dingjie County, Xizang Province, China, June 2020, 2867 m, [CAF *Zhu Xinxin 20200615-1*]	A (0.504, 0.509) B (0.504, 0.514) C (0.511, 0.509)	0.509 ± 0.004
*Populus haoana* var. *megaphylla* C.Wang et Tung^a^	Xianggelila County, Yunnan Province, China, July 2020, 2828 m, [CAF *Zhang Zhongshuai 20200707-2*]	A (0.526, 0.508, 0.515) B (0.530, 0.521, 0.524)	0.521 ± 0.008
*Populus kangdingensis* C. Wang et Tung^a^	Kangding, Sichuan Province, China, July 2020, 3501 m, [CAF *Zhang Zhongshuai 20200721-2*]	A (0.523, 0.518, 0.514) B (0.493, 0.488, 0.496)	0.505 ± 0.015
*Populus koreana* Rehd.^a^	Daqingshan, Heilongjiang Province, China, Sep. 2020, 302 m, [CAF *Zhang Zhongshuai 20200913-13*]	A (0.482, 0.484) B (0.487, 0.492)	0.486 ± 0.004
*Populus lasiocarpa* Oliv.^a^	Wenchuan county, Sichuan Province, China, July 2020, 1750 m, [CAF *Zhang Zhongshuai 20200717-39*]	A (0.515, 0.518) B (0.515, 0.521)	0.517 ± 0.003
*Populus qiongdaoensis* T. Hong & P. Luo^a^	Bawangling, Changjing county, Hainan Province, China, Mar. 2020, 1300 m, [CAF *Shenjun 20200310-1*]	A (0.470, 0.469) B (0.479, 0.472)	0.473 ± 0.005
*Populus rotundifolia* Griff.^a^	Ninglang County, Yunnan Province, China, July 2020, 3082 m, [CAF *Zhang Zhongshuai 20200705-11*]	A (0.486, 0.485)	0.486 ± 0.001
*Populus simonii* Carr.^a^	Luding County, Sichuan Province, China, July 2020, 1815 m, [CAF *Zhang Zhongshuai 20200720-16*]	A (0.488) B (0.486, 0.498)	0.491 ± 0.006
*Populus suaveolens* Fisch.^a^	Baishan, Jilin Province, China, Sep. 2020, 868 m, [CAF *Zhang Zhongshuai 20200909-20*]	A (0.475, 0.481)	0.478 ± 0.004
	Jingyu, Jilin Province, China, Sep. 2020, 586 m, [CAF *Zhang Zhongshuai 20200909-4*]	A (0.703, 0.706)	0.705 ± 0.002
*Populus szechuanica* Schneid.^a^	Baoxing County, Sichuan Province, China, July 2020, 2568 m, [CAF *Zhang Zhongshuai 20200718-35*]	A (0.493, 0.497) B (0.507, 0.504)	0.5 ± 0.006
	Meixian County, Shannxi Province, China, July 2020, 2182 m, [CAF *Zhang Zhongshuai 20200731-11*]	A (0.487, 0.493)	0.49 ± 0.004
	Wenchuan County, Sichuan Province, China, July 2020, 2223 m, [CAF *Zhang Zhongshuai 20200717-47*]	A (0.494, 0.496, 0.501)	0.497 ± 0.004
*Populus wilsonii* Schneid.^a^	Meixian, Shannxi Province, China, August 2020, 2491 m, [CAF *Zhang Zhongshuai 20200801-1*]	A (0.503, 0.507) B (0.501, 0.525) C (0.499, 0.510)	0.508 ± 0.009
*Populus yatungensis* (C. Wang et P. Y. Fu) C. Wang et Tung^a^	Yadong County, Xizang Province, China, June 2020, 2844 m, [CAF *Zhang Zhongshuai 20200616-1*]	A (0.524, 0.531) B (0.525, 0.524) C (0.517, 0.521)	0.524 ± 0.005
*Populus yuana* C. Wang et Tung^a^	Xianggelila County, Yunnan Province, China, July 2020, 2639 m, [CAF *Zhang Zhongshuai 20200706-11*]	A (0.512, 0.509, 0.519)	0.513 ± 0.005
*Populus yunnanensis* var. *pedicellata* C.Wang et Tung^a^	Denqin County, Yunnan Province, China, July 2020, 2097 m, [CAF *Zhang Zhongshuai 20200709-18*]	A (0.514, 0.520) B (0.504, 0.508) C (0.526, 0.526) D (0.513, 0.511, 0.516) E (0.512, 0.512, 0.508) F (0.52, 0.506, 0.517)	0.514 ± 0.007

^a^1 C nuclear DNA amounts of these taxa are reported for the first time

^b^1 C nuclear DNA amounts of these taxa are first reported for the genus they represented

## Discussion

### The taxonomic implications of chromosome number and 1 C DNA amount

The monotypic *Dianyuea* includes *D. turbinate*, which is an enigmatic species endemic to the western Yunnan Province, China. It was first described and placed in the genus *Flacourtia* Comm. ex L’Hér. of Salicoideae as *F. turbinata* H.J. Dong & H. Peng in 2013 [51]. Using plastid *trn*L-F, *mat*K, and *rbc*L sequence, Shang et al. found *F. turbinata* and the genus *Scyphostegia* of Scyphostegioideae form a strongly supported clade which is sister to the crown group of Salicoideae [46]. These indicate that *F. turbinata* does not belong to Salicoideae, and should be separated from *Flacourtia*. In addition, *F. turbinata* has many characteristics which are different from that of *Flacourtia*. For example, male flowers with six stamens connate to form a column (polystemonous and free in *Flacourtia*), basal placentation (parietal in *Flacourtia*), capsule (berry in *Flacourtia*), and fleshy, pubescent, and lobed appendages around seeds (absent in *Flacourtia*) [46]. Previous studies show the chromosome number (CN) of *Flacourtia* is *n* = 10 or 11. Our results show that the CN of *F. turbinata* is 2*n* = 38 or 36 +2B. Among all the 38 chromosomes, there are also two small chromosomes without any visible constriction stably appeared in almost all metaphase cells of *F. turbinata* examined. If consider them as two extra-chromosomes, the CN of *F. turbinate* is alternatively 2*n* = 36 + 2B. In order to provide conclusive evidence, further research is needed. The genome size of *F. turbinata* (4.315 pg) is much bigger than that of *Flacourtia* (0.433-0.568 pg). The chromosome number and genome size give additional evidence for excluding *F. turbinata* from *Flacourtia*.

The monotypic *Scyphostegia* includes *S. borneensis* Stapf, which is endemic to the northern part of Borneo [52]. It has been placed in or considered to be closely related to several different and distantly-related families, including Monimiaceae, Moraceae, Tamaricaceae, and Flacourtiaceae due to its unusual combination of external morphology (dioecy, basal placentation, 3-merous flowers, and telescoping inflorescence bracts) and anatomical features (stem, leaf, flower, and fruit) [52–55]. Shang et al. found a strongly supported sister relationship of *Dianyuea* and *Scyphostegia*, and they are sister to all taxa of Salicoideae [46]. The CN of *Scyphostegia* and *Dianyuea* are 2*n* = 18 and 38 (perhaps 36 + 2B), respectively. They are possibly based on the same basic chromosome number, and a polyploid event probably happened in *Dianyuea*, which is fairly common in Malpighiales [56]. Therefore, our results provide additional evidence for the sister relationship between *Dianyuea* and *Scyphostegia*, which indicate by molecular phylogenetic study [46].

The identity of the sister taxon of *Populus*-*Salix* and the relationship of *Populus*, *Salix*, and their relatives have been long-term discussed and remained controversial. There are two proposed phylogenetic relationships. In the first situation, the two Asia genera *Idesia* and *Bennettiodendron* have closer relationships with *Populus*-*Salix* than *Poliothyrsis*, *Itoa*, and *Carrierea*. This is supported by a phylogenetic study of Malpighiales using 13 gene regions, including 10 plasmid genes and 3 nuclear genes [7]. A similar relationship has been revealed by Xi et al. [8] and Zhang et al. [6] using plastome sequence phylogeny. Besides, the close relationship of *Idesia* with *Populus* and *Salix* was supported by the occurrence of the rust fungus, *Melampsora*, in *Idesia*, *Populus* and *Salix* [9]. In the second situation, the relationships of *Poliothyrsis*, *Itoa*, *Carrierea* and *Populus*-*Salix* are closer than that between *Idesia* and *Populus*-*Salix* as illustrated in Fig. 1. This relationship is supported by the landmark phylogenetic research of Salicaceae which used plastid *rbc*L DNA sequence, and included a comprehensive sampling of Salicaceae [3]. Both evolutionary relationships are mainly based on plasmid sequences, and may be affected by chloroplast capture. Of the 3 subfamilies and 55 genera in Salicaceae, the three works support the first relationship covered 6 genera (2 subfamilies), 11 genera (3 subfamilies), and 11 genera (3 subfamilies), respectively [6–8]. In the second proposed phylogeny, they sampled 22 genera (3 subfamilies). More and more studies show that even if the same gene marker set is used, the difference of taxon sampling density will lead to the contradiction of phylogenetic trees [56]. And the increasing importance of taxon sampling for phylogenetic inference has been proposed and high-lightened [56–58]. Our results in the chromosome number and genome size also provide some hints. The history of genome duplication events in *Populus* provided evidence that the progenitor of *Populus* had a base chromosome number of 10. The salicoid whole-genome duplication (WGD) led to the doubling of chromosome number and the subsequently genome-wide reorganization and joining of chromosomes result in the *n* = 19 chromosome karyotype of *Populus* [59]. *Itoa*, *Poliothyrsis*, and *Carrierea* all share the CN of *n* = 20. Their DNA contents are similar with that of *Populus* and *Salix*. All the DNA C-values of these five genera are ranged from 0.36 to 0.685 pg, and even the polyploidy genomes of *Populus* and *Salix* are less than 1 pg. *Idesia* and *Bennettiodendron* both share the CN of *n* = 21. They also have much bigger DNA C-values up to 1.211 pg and 3.296 pg, respectively. In addition, *Itoa*, *Poliothyrsis*, and *Carrierea* have more morphological similarities with *Populus* and *Salix* than *Idesia* and *Bennettiodendron*, for example, the capsule and winged seeds. Therefore, we adopt the second phylogeny in this study. However, we can’t exclude other possibilities until in-depth analysis with more data and more comprehensive sampling are performed.

### The phylogenetic placement of the salicoid whole-genome duplication

The salicoid WGD event is present in all sequenced poplars and willows [22, 59–64]. The time of salicoid WGD was deduced as 8 to 13 Ma when naively calibrated the molecular clock using synonymous rates observed in the Brassicaceae [59]. However, the WGD event is probably shared by poplars and willows [22, 59], and fossil record shows that the *Populus* and *Salix* lineages diverged 60 to 65 Ma [65, 66]. Thus, the salicoid WGD is placed at or near the lineages diverged time of 60 to 65 Ma [59]. This time point is coincident with the previous hypothesis that multiple WGD events in independent lineages of land plants appear to cluster around the Cretaceous - Paleogene (K-Pg) boundary, around 66 Ma [16]. In addition, the Salicoideae was supposed to split from Scyphostegioideae at 68.9 (78.7-59.8) Ma [8]. In this study, we found species in clade A and B of Salicoideae mainly have *n* = 9, 10, or 11 and all species in clade C have twice or more CNs (Fig. 1). Thus, the occurrence of WGD event in the crown group of Salicoideae clade C is the most parsimony evolutionary scenario. Otherwise, if assume the WGD event in the crown group of the whole Salicoideae, we have to suppose a reduction of ploidy level in clade A and B which is improbability due to failure of homologous pairing in meiosis and the fact that, polyploid abundance is only expected to increase over time, since polyploidization is an irreversible process [67]. If assume several WGD events independently occurred in genera of clade C, genome-wide reconstruction of gene families and molecular clock analysis across these genera are required to confirm that. However, we must take this conclusion carefully before dense sampling of genomic sequence data are investigated.

### The success of poplars and willows

Polyploidy is thought to be a major evolutionary driving force in angiosperm diversification [14]. However, there is often a lag-time or delay between the WGD event and subsequent radiations [68, 69]. In clade C, *Poliothyrsis*, *Itoa*, *Carrierea*, *Idesia*, *Bennettiodendron*, *Olmediella*, *Macrohasseltia* are considered to be sister genera close to *Populus* and *Salix* [4, 7, 8]. It is noteworthy that the position of *Olmediella* and *Macrohasseltia* is uncertain, so they are excluded from the tree in Fig. 1. Altogether, *Poliothyrsis*, *Idesia*, *Olmediella* and *Macrohasseltia* are monotypic, while *Itoa*, *Carrierea*, and *Bennettiodendron* all have two species respectively [70]. For comparison, *Populus* and *Salix* including approximately 22-45 and 330-500 species, respectively [1, 71]. Our results indicate that the WGD may occur in the crown group of clade C. The species richness in *Populus*-*Salix* group and species poverty in their relatives suggest that there is a lag-time for the subsequent radiations after the salicoid WGD event in clade C.

The WGD radiation lag-time model proposed by Schranz et al. suggested that major radiation events are likely not directly driven by the WGDs, but rather by secondary dispersal events triggered by later changing environmental conditions (climate, geological, etc.), evolutionary arms races (e.g. herbivore and plant host), co-radiations (e.g. specialized pollinator and plant host), and migration events into new environments [68]. According to our own observation and literature records, all the seven sister genera of *Populus* and *Salix* tend to have a narrow and fragmentized habitat, and they are common but not key elements of their habitat [70, 72]. The first five genera (*Poliothyrsis*, *Itoa*, *Carrierea*, *Idesia*, and *Bennettiodendron*) are restricted to East Asia and Southeast Asia, and the latter two genera (*Olmediella* and *Macrohasseltia*) are endemic to Central America [4]. They survive in tropical and subtropical forest except for *Idesia* which can reach the southern edge of temperate zone [70]. *Populus* and *Salix* have their maximum species richness in temperate regions of the northern hemisphere and are diversified extensively in high latitude [1]. Many poplars and willows are keystone species in northern hemisphere especially in riparian forest [1].

In conclusion, the radiation and adaptation of *Populus* and *Salix* might be driven by both the WGD and environmental changes. As our prediction, the seven genera in Clade C shared a WGD, which provided sources for adaptation, speciation and evolution. After the WGD, five genera were retained in narrow and fragmentized habitats, while *Populus* and *Salix* migrated to colder environments. A suit of adaptive innovations, including cold tolerance (in almost all poplars and willows), drought tolerance (*Populus euphratica*, *P. alba*, etc.), and plateau adaptability (in *Salix* sect. *Lindleyanae*) enable *Populus*-*Salix* to occupy northern hemisphere temperate area.

## Conclusions

In this study, we report the somatic CN of seventeen species from eight genera in Salicaceae. Of these, CNs for twelve species (*Itoa orientalis*, *Poliothyrsis sinensis*, *Carrierea calycina*, *Bennettiodendron leprosipes*, *Dianyuea turbinata*, *Casearia velutina*, *Populus glauca*, *P. pamirica*, *P. szechuanica*, *P. wilsonii*, *P. yatungensis*, and *P. yunnanensis*) and for five genera (*Itoa*, *Poliothyrsis*, *Carrierea*, *Bennettiodendron*, and *Dianyuea*) are reported for the first time. The CN of *Idesia polycarpa* was confirmed to be 2*n* = 42. The genome sizes of 35 taxa belonging to 14 genera of *Populus* and *Populus*-relatives were estimated. Of these, the genome size of 12 genera (*Dianyuea*, *Homalium*, *Scolopia*, *Xylosma*, *Dovyalis*, *Flacourtia*, *Oncoba*, *Bennettiodendron*, *Idesia*, *Carrierea*, *Poliothyrsis*, and *Itoa*) and of all taxa except *Populus euphratica* are first reported. Our research greatly enriched the basic cytological characteristics of Salicaceae. The variation of CN and genome size across Salicaceae indicate frequent ploidy changes and a widespread sharing of the salicoid whole genome duplication (WGD) by the relatives of *Populus* and *Salix*. The phylogenetic asymmetry in clade of *Populus*, *Salix*, and their close relatives suggests that there is a lag-time for the subsequent radiations after the salicoid WGD event. Our results provide useful data for studying the evolutionary events of Salicaceae.

## Methods

### Taxon sampling and identification

**Fig. 4:**
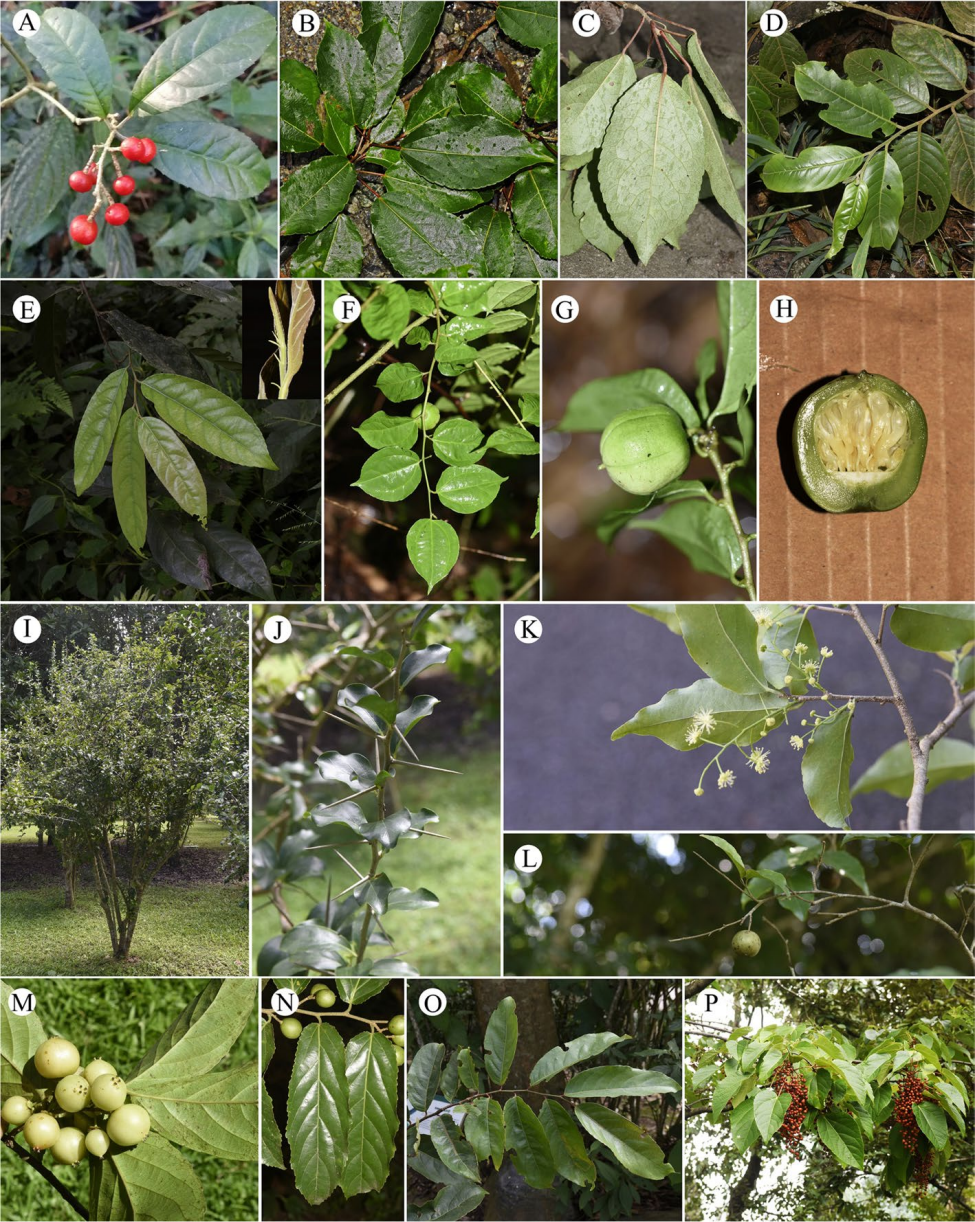
Morphological features of Salicaceae (I). (**A**) *Bennettiodendron leprosipes*, (**B**-**C**) *Carrierea calycina*, (**D**) *Casearia velutina*, (**E**) *C. graveolens*, (**F**-**H**) *Dianyuea turbinate*, (**I**-**J**) *Dovyalis caffra*, (**K**-**L**) *Flacourtia indica*, (**M**) *F. inermis*, (**N**) *F. rukam*, (**O**) *Homalium ceylanicum*, and (**P**) *Idesia polycarpa*

**Fig. 5:**
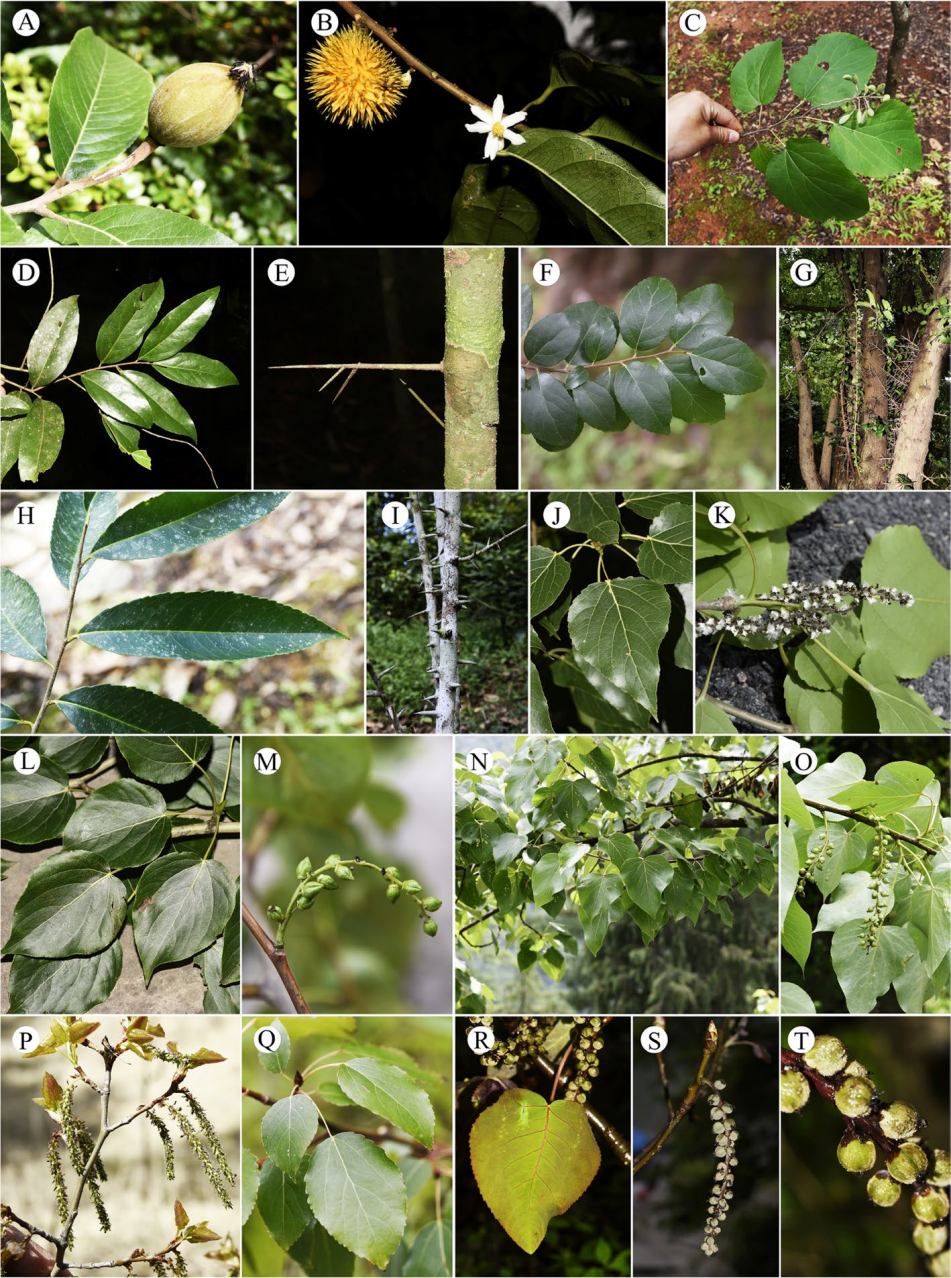
Morphological features of Salicaceae (II). (**A**) *Itoa orientalis*, (**B**) *Oncoba echinate*, (**C**) *Poliothyrsis sinensis*, (**D**-**E**) *Scolopia chinensis*, (**F**-**G**) *Xylosma congesta*, (**H**-**I**) *X. longifolia*, (**J**-**K**) *Populus adenopoda*, (**L**-**M**) *P. cathayana*, (**N**-**O**) *P. ciliate*, (**P**-**Q**) *P. davidiana*, and (**R**-**T**) *P. glauca*

**Fig. 6:**
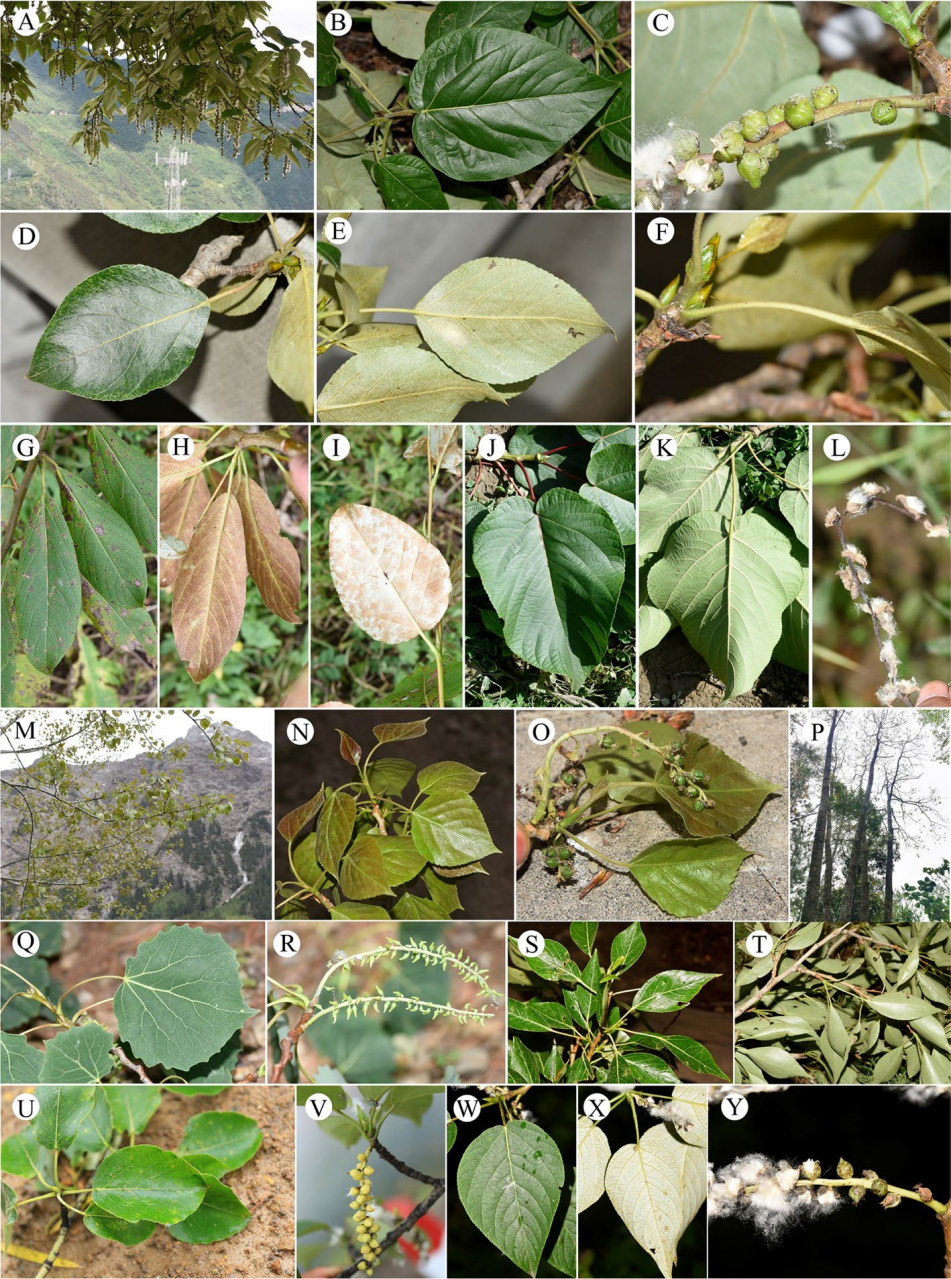
Morphological features of Salicaceae (III). (**A**-**C**) *Populus haoana* var. *megaphylla*, (**D**-**F**) *P. kangdingensis*, (**G**-**I**) *P. koreana*, (**J**-**L**) *P. lasiocarpa*, (**M**-**O**) *P. pamirica*, (**P**) *P. qiongdaoensis*, (**Q**-**R**) *P. rotundifolia*, (**S**-**T**) *P. simonii*, (**U**-**V**) *P. suaveolens*, and (**W**-**Y**) *P. szechuanica*

**Fig. 7:**
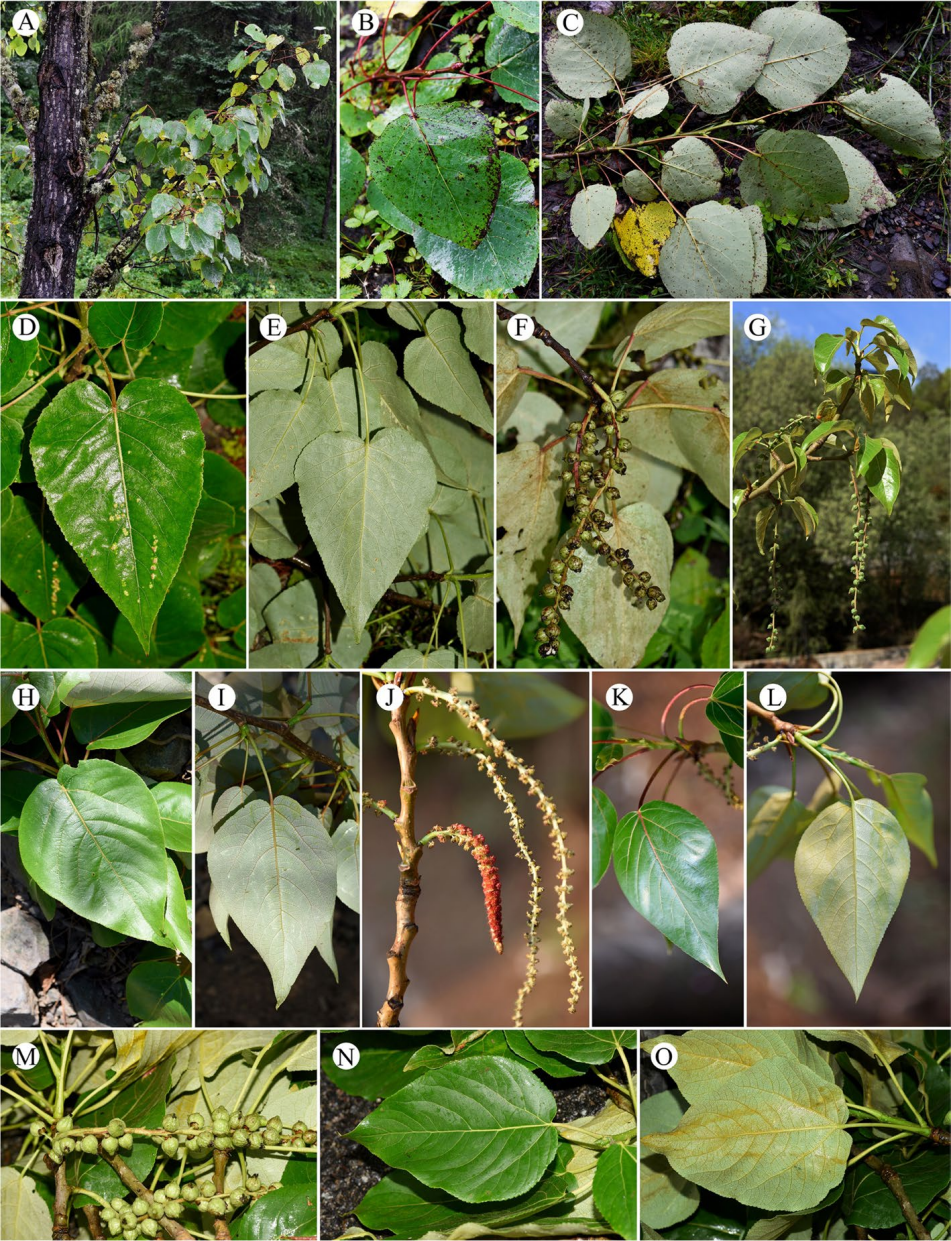
Morphological features of Salicaceae (IV). (**A**-**C**) *Populus wilsonii*, (**D**-**F**) *P. yatungensis*, (**G**-**I**) *P. yuana*, (**J**-**L**) *P. yunnanensis*, and (**M**-**O**) *P. yunnanensis* var. *pedicellata*

In this study, we included 17 taxa from eight genera for cytological analysis (Table 1) and 35 taxa from fourteen genera for genome size estimation (Table 2). Individuals were collected from field work by Zhong-Shuai Zhang in the vast area of China conducted in 2020 and 2021, as well as Xishuangbanna Tropical Botanical Garden and Kunming Botanical Garden in China (Tables 1 and 2). Where possible, more than one and up to six individuals were included per taxon. Sampled individuals were identified by all the authors according to appropriate literatures, and type materials from different herbariums. Due to the taxonomy of *Populus* is still in heated debate and there is no recent taxonomic revisions of many genera studied in this article. We give all the sampling site and relevant pictures to aid identification (Figs. 4, 5, 6 and 7). The voucher specimens of the studied materials are all preserved in the herbarium of Chinese Academy of Forestry (CAF) [73]. The source numbers of voucher specimens are listed in Tables 1 and 2.

### Cytological analysis

Branch cuttings or seedlings of samples were collected and planted in flowerpots in the greenhouse at 25℃. Vigorous root tips were pre-treated with ice-water mixture in dark room for 24 h. After incubation, the tips were fixed in Carnoy I solution (3:1 ethanol: glacial acetic acid) at 4 °C for at least 3 h. They were then digested at 37 °C in a combination (1:1) of 2 % cellulase and 2 % pectinase for 30 to 60 min before staining with an improved carbol-fuchsin solution and squashed for cytological observation [74]. Standard liquid nitrogen method was used to make permanent slides that were preserved at Chinese Academy of Forestry. The photo micrographs were taken using an Axio Imager A1 microscope (Zeiss, Germany). Only complete cells with clear outline and scattered chromosomes were selected for observation. The chromosome number of each taxon was determined by checking multiple random selected mitosis metaphase cells of individuals. We detected more than 5, up to 42 (in *Dianyuea turbinata*) cells of root tips of each taxon, and determined the chromosome number only when all cells showed the same count.

### Flow cytometry

The fresh leaves of the majority individuals were collected from the transplanted plants cultivated in greenhouse. These leaves were kept on ice and used for flow cytometer analysis within 12 h. The materials from Xishuangbanna Tropical Botanical Garden and Kunming Botanical Garden were collected directly from the garden trees. For botanical garden materials, populations from different origin are labeled by resource numbers. The materials were stored at 0 ℃ immediately and measured within three days. One to three technical repetitions were tested according to the availability of materials. We used internal standards for all measurements and the internal standards were selected based on appropriate non-overlapping genome size. Fresh leaves of *Zea mays* L. B73 (2.425 pg/1 C) were used as internal standards for *Dianyuea turbinata* and *Idesia polycarpa* [75]. Fresh leaves of *Glycine max* (L.) Merr. Williams 82 (1.155 pg/1 C) were used as internal standards for the rest samples [76]. Approximately 0.5 cm^2^ leaf of the standard and samples were co-chopped with a sharp razor blade for ca. 10 to 20 s in a Petri dish containing 0.25 mL ice-cold nuclei extracting buffer (30 mmol/L Na_3_C_6_H_5_O_7_·2H_2_O, 45 mmol/L MgCl_2_, 20 mmol/L MOPS, 20 mmol/L NaCl, 20 mmol/L EDTA-Na_2_, 0.1 % volume percentage Triton X-100, 0.5 % volume percentage Tween-20, 1 % volume percentage PVP, pH=7.0). The nuclei extracting buffer is slightly modified from Galbraith’s buffer and was preserved at 4℃ until use [77]. The homogenate was gently sucked up by pipette and passed through 48 μm nylon mesh filters into 5 mL plastic round-bottom Falcon tubes (Corning, New York, N.Y., USA). A volume of 0.5 ml staining buffer (CyStain PI Absolute P, Sysmex Partec GmbH Görlitz, Germany), 3 µl propidium iodide (CyStain PI Absolute P, Sysmex Partec GmbH Görlitz, Germany) and 1.5 µl RNaseA were added and mixed by gentle shaking. Samples were incubated with the staining solution on ice for 15 min in darkness prior to flow cytometry analysis. The homogenates were analyzed based on light scatter and fluorescence signals produced from 20 mW laser illumination at 488 nm using a BD LSRFortessa^TM^ cell analyzer (BD Biosciences, Franklin Lakes, NJ). At least 3 × 10^3^ nuclei were collected in each measurement. Data were collected and analyzed by BD FACSDiva 7.0 (BD Biosciences, Franklin Lakes, NJ). The coefficient of variation among nuclei (CVn) was calculated as follow: CVn = SD/M, where SD was the standard deviation of the nuclei distribution, and M was the mean channel number [78]. We performed a pre-analyze on some samples of *Populus* and all samples of the other genera. We collected the PI fluorescence intensity of each sample without internal standard and checked whether there were several peaks arranged in an endoreplication fashion first. If endoreplication exists, there will be additional peaks with 8 C, 16 C, 32 C and even higher DNA levels besides the 2 C (G1) and 4 C (G2) peaks (Response Fig. 1 A). As a result, we did not find any polyploidization peaks of DNA. Then, we preformed analysis on samples with internal standard, and used the two large peaks representing G1 nuclei of the reference and the sample to estimate the DNA contents. The formula 1 C DNA content (pg) of the standard × average of sample G1 peak / average of standard G1 peak was used to estimate the 1 C DNA content of sample cells at G1 phase.

## Supplementary Information

**Additional file 1.**

**Additional file 2.**

## Data Availability

All data generated or analyzed during this study are included in this published article and its supplementary information files. The voucher specimens of the materials analyzed during the current study are preserved in the herbarium of Chinese Academy of Forestry (CAF) and available from the corresponding author on reasonable request.
