# Supplementary material for: Frequent ploidy changes in Salicaceae indicates widespread sharing of the salicoid whole genome duplication by the relatives of Populus L. and Salix L
Source: BMC Plant Biol. 2021 Nov 13;21:535. doi: 10.1186/s12870-021-03313-x (PMC8590345; doi:10.1186/s12870-021-03313-x)
Supplement: Supplementary file 1 — Additional file 1. [file 12870_2021_3313_MOESM1_ESM.doc]

Table S1. The chromosome number of Salicaceae obtained from Chromosome Counts Database (CCDB, version 1.58) (http://ccdb.tau.ac.il/).

| Latin name | Chromosome number | Original reference |
| --- | --- | --- |
| *Casearia graveolens* Dalzell | n = 42 | Mehra 1976 |
| *Casearia tomentosa* Roxb. | n = 21; 2n = 42 | Mehra 1976; Krishnan 1977 |
| *Casearia barteri* Mast. | 2n = 44 | Cave 1960 |
| *Casearia nigrescens* Tul. | n = 21 | Bir et al. 1982 |
| *Scyphostegia borneensis* Stapf | n = 9; 2n = 18 | Hou 1972 |
| *Abatia parviflora* Ruiz & Pav. | n = c.36 | Cave 1964 |
| *Azara serrata* var. *fernandeziana* (Gay) Reiche | n = 9 | Sanders et al. 1983 |
| *Dovyalis abyssinica* (A. Rich.) Warb. | 2n = 20 | Arends 1978 |
| *Flacourtia indica* (Burm.) Merr. | n = 11 | Bir et al. 1982 |
| *Flacourtia inermis* Roxb. | 2n = 20 | Krishnan 1977 |
| *Flacourtia montana* J. Graham | n = 10 | Löve & Löve 1982 |
| *Flacourtia jangomas* (Lour.) Raeusch. | n = 11 | Bir et al. 1982 |
| *Homalium ceylanicum* (Gardner) Benth. | 2n = 20 | Krishnan 1977 |
| *Homalium bhamoense* Cubitt & W. W. Sm. | n = 11 | Löve 1979 |
| *Idesia polycarpa* Maxim. | ca. 2n = 44 | Corti 1948 |
| *Oncoba brachyanthera* Oliv. | 2n = 20 | Mangenot & Mangenot G. 1957 |
| *Oncoba spinosa* Forssk. | n = 11 | Löve 1980; Singhal et al. 1985 |
| *Oncoba dentata* Oliv. | 2n = 48 | Mangenot & Mangenot G. 1962; Gadella 1972. |
| *Populus adenopoda* Maxim. | n = 19 | Smith 1943 |
| *Populus alba* L. | 2n = 38 | Peto 1938; Zhang et al. 2005 |
| *Populus alba* var. *pyramidalis* Bunge | 2n = 38 | Zhang et al. 2005 |
| *Populus angustifolia* E. James | 2n = 38 | Smith 1943 |
| *Populus balsamifera* L. | 2n = 38 | Löve & Löve 1982 |
| *Populus beijingensis* W. Y. Hsu | 2n = 38 | Zhang et al. 2005 |
| *Populus cathayana* Rehder | 2n = 38 | Qi et al. 2004 |
| *Populus ciliata* Wall. ex Royle | n = 19 | Löve 1980 |
| *Populus deltoides* Marshall | 2n = 38 | Smith 1943 |
| *Populus deltoides* subsp. *wislizenii* (S. Watson) Eckenw. | 2n = 38 | Gervais et al. 1999 |
| *Populus euphratica* Oliv. | 2n = 38 | Chen et al. 2005 |
| *Populus fremontii* S. Watson | 2n = 38 |  |
| *Populus grandidentata* Michx. | 2n = 38 | Smith 1943 |
| *Populus koreana* Rehder | 2n = 38 | Zhang et al. 2005 |
| *Populus lasiocarpa* Oliv. | 2n = 38 | Smith 1943; Chen et al. 2005 |
| *Populus laurifolia* Ledeb. | 2n = 38 | Smith 1943 |
| *Populus nigra* L. | 2n = 38; 57 | Pogan et al. 1982 |
| *Populus nigra* var. *italica* Münchh. | 2n = 38 | Zhang et al. 2005 |
| *Populus pseudosimonii* Kitag. | 2n = 38 | Qi et al. 2004; Zhang et al. 2005 |
| *Populus simonii* Carrière | 2n = 38 | Smith 1943; Chen et al. 2005; Zhang et al. 2005 |
| *Populus suaveolens* Fisch. ex Loudon | 2n = 38 | Zhang et al. 2005 |
| *Populus tremula* L. | 2n = 38 | Smith 1943; Zhang et al. 2005 |
| *Populus tremuloides* Michx. | 2n = 38 | Smith 1943 |
| *Populus trichocarpa* Torr. & A. Gray ex Hook. | 2n = 38 | Qi et al. 2004; Zhang et al. 2005 |
| *Populus* × *canadensis* Moench | 2n = 38 | Zhang et al. 2005 |
| *Populus* × *canescens* (Aiton) Sm. | 2n = 38; 57 | Peto 1938 |
| *Populus* × *hopeiensis* Hu & H. F. Chow | 2n = 38 | Zhang et al. 2005 |
| *Populus* × *jackii* Sarg. | n = 19 | Eckenwalder 1984 |
| *Populus* × *parryi* Sarg. | n = 19 | Eckenwalder 1984 |
| *Populus* × *xiaozhuanica* W. Y. Hsu & Liang | 2n = 38 | Zhang et al. 2005 |
| *Prockia crucis* P. Browne ex L. | 2n = 18 | Morawetz 1981 |
| *Salix abscondita* Laksch. | 2n = 38 | Aguilera et al. 2011 |
| *Salix acmophylla* Boiss. | 2n = 38 | Löve 1969 |
| *Salix acutifolia* Willd. | 2n = 38 | Blackburn & Harrison 1924 |
| *Salix aegyptiaca* L. | 2n = 38 | Håkansson 1955 |
| *Salix alaxensis* (Andersson) Coville | 2n = 38 | Suda & Argus 1969 |
| *Salix alba* L. | 2n = 38 | Suda & Argus 1968 |
| *Salix alba* var. *vitellina* (L.) Stokes | 2n = 76 | Dobes et al. 1997 |
| *Salix alpina* Scop. | 2n = 38 | Dobes et al. 1997 |
| *Salix amygdaloides* Andersson | 2n = 38 | Suda & Argus 1968 |
| *Salix angustifolia* Cariot | 2n = 38 | Rice et al. 2015 |
| *Salix apennina* A. K. Skvortsov | 2n = 114 | Neumann & Polatschek 1972 |
| *Salix appendiculata* Vill. | 2n = 38, 114 | Neumann & Polatschek 1972; Dobes et al. 1997 |
| *Salix appenina* Skvortsov | 2n = 55, 57, 59 | Büchler 1985 |
| *Salix arbuscula* L. | 2n = 38 | Harrison 1926 |
| *Salix arbusculoides* Andersson | 2n = 38 | Suda & Argus 1969 |
| *Salix arctica* Pall. | 2n = 76, 114 | Suda & Argus 1969 |
| *Salix arctophila* Cockerell | 2n = 76 | Löve 1982 |
| *Salix argyracea* E. L. Wolf | 2n = 76 | Rice et al. 2015 |
| *Salix argyrocarpa* Andersson | 2n = 76 | Rice et al. 2015 |
| *Salix arizonica* Dorn | n = 19 | Marhold 2006 |
| *Salix athabascensis* Raup | 2n = 76, 114 | Suda & Argus 1969 |
| *Salix athabascensis* Raup | 2n = 76, 114 | Dorn 1975; Löve & Löve 1982 |
| *Salix atrocinerea* Brot. | 2n = 76 | Neumann & Polatschek 1972 |
| *Salix aurita* L. | 2n = 38, 76 | Blackburn & Harrison 1924 |
| *Salix babylonica* L. | 2n = 38, 76, 114 | Ma et al. 1990; Zhang 1998 |
| *Salix bakko* Kimura | 2n = 38, 76 | Rice et al. 2015 |
| *Salix barclayi* Andersson | 2n = 76 | Rice et al. 2015 |
| *Salix bebbiana* Sarg. | 2n = 38 | Dorn 1975 |
| *Salix bicolor* Ehrh. ex Willd. | 2n = 38; 57; 114 | Büchler 1985; Amorim et al. 2012 |
| *Salix boganidensis* Trautv. | 2n = 38 | Yurtsev 1982 |
| *Salix bonplandiana* Kunth | 2n = 42 | Darlington & Wylie 1955 |
| *Salix boothii* Dorn | 2n = 76 | Rice et al. 2015 |
| *Salix borealis* Fr. | 2n = 152 | Darlington & Wylie 1955 |
| *Salix brachycarpa* Nutt. | 2n = 38 | Suda & Argus 1968 |
| *Salix brachycarpa* Nutt. | 2n = 38 | Löve & Löve 1982. |
| *Salix breviserrata* Flod. | 2n = 38 | Büchler 1985 |
| *Salix caesia* Vill. | 2n = 76 | Dobes et al. 1997 |
| *Salix calodendron* Gand. | 2n = 76 | Rice et al. 2015 |
| *Salix candida* Flüggé ex Willd. | 2n = 38 | Suda & Argus 1968 |
| *Salix caprea* L. | 2n = 38 | Löve 1979 |
| *Salix caspica* Pall. | 2n = 38 | Zakirova 1999 |
| *Salix chaenomeloides* Kimura | 2n = 38 | Rice et al. 2015 |
| *Salix chamissonis* Andersson | 2n = 114 | Zhukova 1980 |
| *Salix chilkoana* Sukaczev | 2n = 57 | Rice et al. 2015 |
| *Salix chlorolepis* Fernald | 2n = 38 | Rice et al. 2015 |
| *Salix cinerea* L. | 2n = 44, 76 | Dobes et al. 1997; Zakirova 1999 |
| *Salix columbiana* Argus | 2n = 38 | Rice et al. 2015 |
| *Salix commutata* Bebb | 2n = 38 | Rice et al. 2015 |
| *Salix cordata* Michx. | 2n = 38; 44 | Dorn 1995; Darlington & Wylie 1955 |
| *Salix crataegifolia* Bertol. | 2n = 38 | Büchler 1985 |
| *Salix daltoniana* Andersson | 2n = 38 | Rice et al. 2015 |
| *Salix daphnoides* Vill. | 2n = 38, 57, 152 | Löve 1969; Wilkinson 1944; Dobes et al. 1997 |
| *Salix darpirensis* Jurtzev & Khokhrj. | 2n = 76 | Petrovsky & Zhukova 1983 |
| *Salix discolor* Andersson | 2n = 38 | Rice et al. 2015 |
| *Salix discolor* Muhl. | 2n = 76 | Suda & Argus 1968 |
| *Salix drummondiana* Barratt ex Hook. | 2n = 38, 57, 76 | Dorn 1975; Suda & Argus 1968 |
| *Salix eastwoodiae* Cockerell ex A. Heller | 2n = 76 | Dorn 1975 |
| *Salix eleagnos* Scop. | 2n = 38 | Druskovic 1995 |
| *Salix eriocephala* Michx. | 2n = 38, 44 | Dorn 1995; Wilkinson 1944 |
| *Salix exigua* Nutt. | 2n = 38 | Suda & Argus 1968 |
| *Salix famelica* (C. R. Ball) Argus | 2n = 38 | Dorn 1995 |
| *Salix fimbriata* Czerep. | 2n = 38 | Zhukova 1973 |
| *Salix floridana* Chapm. | 2n = 38 | Rice et al. 2015 |
| *Salix foetida* Schleich. | 2n = 38 | Neumann & Polatschek 1972 |
| *Salix fragilis* Forssk. | 2n = 38, 76 | Majovsky 1974; Vachova 1978 |
| *Salix fragilis* Forssk. | 2n = 38, 76, 114 | Darlington & Wylie 1955; Druskovic 1995. |
| *Salix fuscescens* Andersson | 2n = 38 | Suda & Argus 1969 |
| *Salix geyeriana* Andersson | 2n = 38 | Dorn 1975 |
| *Salix gilgiana* Seemen | 2n = 76 | Suda 1963 |
| *Salix glabra* Scop. | 2n = 38, 76 | Dobes et al. 1997; Druskovic 1995 |
| *Salix glauca* L. | 2n = 76, 114 | Suda & Argus 1969 |
| *Salix gmelinii* Pall. | 2n = 57, 76, 114 | Neumann & Polatschek 1972 |
| Salix gooddingii C. R. Ball | n = 19 | Raven et al. 1965 |
| Salix gracilistyla Miq. | 2n = 38 | Rudyka 1990 |
| Salix hastata L. | 2n = 35, 37, 38, 39 | Büchler 1985 |
| Salix hegetschweileri Heer | 2n = 67, 73, 76, 78, 81, 84 | Büchler 1985; Neumann & Polatschek 1972 |
| Salix helvetica Vill. | 2n = 38 | Büchler 1985 |
| Salix herbacea L. | 2n = 38 | Löve & Löve 1982 |
| Salix hexandra Ehrh. | n = 38 | Büchler 1985 |
| Salix hookeriana Barratt ex Hook. | 2n = 114 | Rice et al. 2015 |
| Salix hultenii Flod. | n = 19 | Sun et al. 1996 |
| Salix humboldtiana Willd. | 2n = 38 | Darlington & Wylie 1955 |
| Salix humilis Marshall | 2n = 38 | Suda & Argus 1968 |
| Salix integra Thunb. | 2n = 38 | Aguilera et al. 2011 |
| Salix interior Rowlee | 2n = 38 | Suda & Argus 1968 |
| Salix irrorata Andersson | 2n = 37-39 | Büchler 1986 |
| Salix japonica Thunb. | 2n = 38 | Darlington & Wylie 1955 |
| Salix kamtschatica Vorosch. | 2n = 38 | Probatova 1995 |
| Salix kangensis Nakai | 2n = 38 | Rice et al. 2015 |
| Salix krylovii E. L. Wolf | 2n = 38 | Zhukova 1967 |
| Salix laggeri Wimm. | 2n = 56-58, 76 | Büchler 1986; Neumann & Polatschek 1972 |
| Salix lanata L. | 2n = 38 | Rice et al. 2015 |
| Salix lapponum L. | n = 19 | Büchler 1986 |
| Salix lasiandra Benth. | 2n = 76 | Darlington & Wylie 1955 |
| Salix lasiandra var. caudata (Nutt.) Sudw. | 2n = 76 | Dorn 1975 |
| Salix lasiolepis Benth. | n = 38 | Raven et al. 1965 |
| Salix lemmonii Bebb | 2n = 76 | Dorn 1975 |
| Salix ligulifolia (C. R. Ball) C. R. Ball ex C. K. Schneid. | n = 38 | Dorn 1975 |
| Salix lucida Muhl. | 2n = 76 | Löve & Löve 1982 |
| Salix lutea Nutt. | 2n = 38 | Suda & Argus 1968 |
| Salix maccalliana Rowlee | 2n = ca. 224 | Suda & Argus 1968 |
| Salix medemii Boiss. | 2n = 76 | Darlington & Wylie 1955 |
| Salix mielichhoferi Saut. | 2n = 114 | Dobes et al. 1997 |
| Salix mollissima Ehrh. | 2n = 38 | Rice et al. 2015 |
| Salix monticola Bebb | 2n = 38 | Suda & Argus 1968 |
| Salix myrsinites L. | 2n = 38, 190 | Darlington & Wylie 1955 |
| Salix myrtillifolia Andersson | 2n = 38 | Suda & Argus 1968 |
| Salix myrtilloides L. | 2n = 38 | Dobes et al. 1997 |
| Salix myrtilloides Willd. | 2n = 38 | Darlington & Wylie 1955 |
| Salix nigra Marshall | 2n = 38 | Dorn 1975 |
| Salix niphoclada Rydb. | 2n = 38, 76 | Petrovsky & Zhukova 1983 |
| Salix nipponica Franch. & Sav. | 2n = 38 | Aguilera et al. 2011 |
| Salix nivalis Hook. | 2n = 38 | Löve et al. 1971 |
| Salix nummularia Andersson | 2n = 38 | Petrovsky & Zhukova 1983 |
| Salix ovalifolia Trautv. | 2n = 38 | Suda & Argus 1969 |
| Salix pedicellaris Pursh | 2n = 57 | Suda & Argus 1969 |
| Salix pellita (Andersson) Bebb | 2n = 76 | Suda & Argus 1968 |
| Salix peloritana Presl x | 2n = 34, 38 | Büchler 1985 |
| Salix pentandra L. | 2n = 76 | Dobes et al. 1997 |
| Salix petiolaris Sm. | 2n = 38 | Suda & Argus 1968 |
| Salix petiolaris Sm. | 2n = 38 | Suda & Argus 1968 |
| Salix petrophila Rydb. | 2n = 76 | Löve et al. 1971 |
| Salix phlebophylla Andersson | 2n = 38 | Suda & Argus 1969 |
| Salix phylicifolia L. | 2n = 76, 88, 114 | Löve 1977 |
| Salix pierotii Miq. | 2n = 38 | Aguilera et al. 2011 |
| Salix planifolia Pursh subsp. planifolia | 2n = 57, 76 | Suda & Argus 1968; 1969 |
| Salix planifolia subsp. pulchra (Cham.) Argus | 2n = 76 | Suda & Argus 1969 |
| Salix polaris Wahlenb. | 2n = 76, 90,114 | Borgen & Elven 1983; Petrovsky & Zhukova 1983 |
| Salix pseudomonticola C. R. Ball | 2n = 38 | Dorn 1975 |
| Salix pseudomyrsinites Andersson | 2n = 76 | Petrovsky & Zhukova 1983 |
| Salix pseudopentandra (Flod.) Flod. | 2n = 76 | Rice et al. 2015 |
| Salix psilostigma Andersson | n = 19 | Löve 1969 |
| Salix pulchra Cham. | 2n = 76 | Petrovsky & Zhukova 1983 |
| Salix purpurea L. | 2n = 38 | Dobes et al. 1997 |
| Salix pyrenaica Gouan | n = 19 | Amorim et al. 2012 |
| Salix pyrifolia Andersson | 2n = 38 | Löve & Löve 1982 |
| Salix quercifolia Sennen | 2n = 57 | Rice et al. 2015 |
| Salix recurvigemmis A. K. Skvortsov | 2n = 38 | Petrovsky & Zhukova 1983 |
| Salix reinii Franch. & Sav. ex Seemen | n = 76 | Rice et al. 2015 |
| Salix repens L. | 2n = 38 | Rice et al. 2015 |
| Salix reptans Rupr. | 2n = 38 | Petrovsky & Zhukova 1983 |
| Salix reticulata L. | 2n = 38 | Suda & Argus 1969 |
| Salix retusa L. | 2n = 76, 114, 152 | Löve 1976; Dobes et al. 1997 |
| Salix rhamnifolia Pall. | 2n = 38 | Baltisberger et al. 2010 |
| Salix rorida Laksch. | 2n = 38 | Aguilera et al. 2011 |
| Salix rosmarinifolia L. | 2n = 38 | Dobes et al. 1997 |
| Salix rotundifolia Trautv. | 2n = 38, 114 | Petrovsky & Zhukova 1983 |
| Salix rotundifolia var. dodgeana (Rydb.) E. Murray | 2n = 38, 114 | Rice et al. 2015 |
| Salix rubra J. Walker | 2n = 38 | Rice et al. 2015 |
| Salix rupifraga Koidz. | 2n = 38 | Cave 1964. |
| Salix salviifolia Brot. | 2n = 38 | Rice et al. 2015 |
| Salix saxatilis Turcz. ex Ledeb. | 2n = 38, 76, 114 | Petrovsky & Zhukova 1983 |
| Salix schwerinii E.L. Wolf | 2n = 38 | Aguilera et al. 2011 |
| Salix scouleriana Barratt ex Hook. | 2n = 76 | Suda & Argus 1968 |
| Salix seringeana Lecoq & Lamotte | 2n = 38 with 0 to 4 B chromosomes | Darlington & Wylie 1955 |
| Salix serissima (Bailey) Fernald | 2n = 76 | Löve & Löve 1982 |
| Salix serpyllifolia Scop. | 2n = 36, 38, 40, 41 | Büchler 1986 |
| Salix setchelliana C. R. Ball | 2n = 38 | Rice et al. 2015 |
| Salix sieboldiana Blume | 2n = 76 | Darlington & Wylie 1955 |
| Salix silesiaca Willd. |  | Izmailow 1980. |
| Salix silicicola Raup | 2n = 38 | Suda & Argus 1968 |
| Salix sitchensis Sanson ex Bong. | 2n = 38 | Löve 1976 |
| Salix siuzevii Seemen | 2n = 38 | Aguilera et al. 2011 |
| Salix sphenophylla A. K. Skvortsov | 2n = 38, 52-64 | Petrovsky & Zhukova 1983 |
| Salix starkeana Willd. | 2n = 44 | Darlington & Wylie 1955 |
| Salix stolonifera Coville | 2n = 114, 90 | Petrovsky & Zhukova 1983 |
| Salix subcoerulea Piper | 2n = 57 | Suda & Argus 1968 |
| Salix taraikensis Kimura | 2n = 38 | Aguilera et al. 2011 |
| Salix tetrasperma Roxb. | 2n = 38, 76 | Löve 1969. |
| Salix triandra L. | 2n = 38, 44, 57, 88 | Dobes et al. 1997; Wilkinson 1944 |
| Salix tschuktschorum A. K. Skvortsov | 2n = 114 | Petrovsky & Zhukova 1983 |
| Salix turanica Nasarow | 2n = 38 | Löve 1976. |
| Salix udensis Trautv. & C. A. Mey. | 2n = 38 | Petrovsky & Zhukova 1983 |
| Salix uva-ursi Pursh | 2n = 38 | Rice et al. 2015 |
| Salix vestita Pursh | 2n = 38 | Löve & Löve 1982 |
| Salix viminalis L. | 2n = 38, 76 | Thibault 1998 |
| Salix vulpina Andersson | n = 19 | Rice et al. 2015 |
| Salix waldsteiniana Willd. | 2n = 38 | Dobes et al. 1997; Neumann & Polatschek 1972 |
| Salix wolfii Bebb | 2n = 38 | Dorn 1975 |
| Salix × peloritana Prestandr. ex Tineo | 2n = 38 | Büchler 1985 |
| Salix × smithiana Willd. | 2n = 38 | Löve 1976 |
| Scolopia crenata Clos | 2n = 22 | Krishnan 1977 |
| Xylosma controversa Clos | n = 10 | Löve 1977 |
| Xylosma longifolia Clos | n = 10; 2n = 20 | Mehra 1976; Krishnan 1977 |
| Xylosma hawaiensis Seem. | n = 10 | Rice et al. 2015 |

Aguilera PM, Daviña JR, Honfi AI. 2011. IAPT/IOPB chromosome data 12. Taxon. 60: 1784-1796.

Amorim BS, Alves M, Guerra M, Souza LGR. 2012. IAPT/IOPB chromosome data 14. Taxon 61: 1336-1345

Arends JC. 1978. In: N. Halle & de Wilde JJ, *Trichostephanus acuminatus* Gilg (Flacourtiacees), une approche biosystematique. Adansonia 18: 167-182.

Baltisberger M, Kocyan A, Chepinoga VV, Gnutikov AA, Enushchenko IV. 2010. IAPT/IOPB chromosome data 9. Taxon 59: 1298-1302.

Bir SS, Gill BS, Bedi YS, Singhal VK. 1982. Evolutionary status of the woody taxa of Garhwal Himalaya. In Khosla PK, ed. Improvement of forest biomass. Indian Society of Tree Scientists, Solan. 81-96.

Blackburn KB, Harrison JWH. 1924. A preliminary account of the chromosomes and chromosome behaviour in the Salicaceae. Annals of Botany 38: 361-378.

Borgen L, Elven R. 1983. Chromosome numbers of flowering plants from northern Norway and Svalbard. Nordic Journal of Botany 3: 301-306.

Büchler W. 1985. Neue Chromosomenzählungen in der Gattung *Salix*. Botanica Helvetica 95: 165-175.

Büchler W. 1986. Neue Chromosomenzählungen in der Gattung *Salix* II. Botanica helvetica 96: 135-143.

Cave MS. 1960. Index to Plant Chromosome Numbers for 1959. California Botanical Society, Berkeley.

Cave MS. 1964. Index to Plant Chromosome Numbers for 1963. California Botanical Society, Berkeley.

Chen CB, Zhang SG, Li XL, Han SY, Song WQ, Qi LW. 2005. A comparative study on the karyotypes among sections of *Populus*. Guihaia 25: 338-340.

Corti R. 1948. Sul dioicismo di *Idesia polycarpa* Maxim. Plant Biosystem 55: 446-496.

Darlington CD, Wylie AP. 1955. Chromosome atlas of flowering plants. London,

Dobes CH, Hahn B, Morawetz W. 1997. Chromosomenzahlen zur gefässpflanzen-flora Österreichs. Linzer biologische Beiträge 29: 5-43.

Dorn RD. 1975. Cytological and taxonomic notes on North American *Salix*. Madroño 23: 99.

Dorn RD. 1995. A taxonomic study of *Salix* section *Cordatae* subsection *Luteae* (Salicaceae). Brittonia 47: 160-174.

Druskovic B. 1995. IOPB chromosome data 9. International Organization of Plant Biosystematists. Newsletter 24: 15-19.

Eckenwalder JE. 1984. Natural intersectional hybridization between North American species of *Populus* (Salicaceae) in sections *Aigeiros* and *Tacamahaca*. II. Taxonomy. Canadian Journal of Botany 62: 325-335.

Gadella TWJ. 1972. Cytological studies on some flowering plants collected in Africa. Bulletin du Jardin botanique national de Belgique/Bulletin van de Nationale Plantentuin van Belgie 393-402.

Gervais C, Trahan R, Gagnon J. 1999. IOPB chromosome data 14. International Organization of Plant Biosystematics [sic]. Newsletter. 30: 10-15.

Håkansson A. 1955. Chromosome numbers and meiosis in certain Salices. Hereditas 41: 454-482.

Harrison JWH. 1926. Heterochromosomes and polyploidy. Nature 117: 50-50.

Hou D. 1972. Germination, seedling, and chromosome number of *Scyphostegia borneensis* Stapf (Scyphostegiaceae). Blumea 20: 88-92.

Izmailow R. 1980. Cytological studies in *Salix* L. Acta biologica Cracoviensia. Series: Botanica 22: 101-111.

Krishnan N. 1977. Cytotaxonomical studies on Bixaceae and Samydaceae from south India with collected evidences from palynology, anatomy and biochemistry. Ph.D. Thesis, Annamalai University.

Löve Á, Löve D, Kapoor BM. 1971. Cytotaxonomy of a century of Rocky Mountain orophytes. Arctic and Alpine Research 3: 139-165.

Löve A, Löve D. 1982. IOPB chromosome number reports LXXVII. Taxon 31: 761-777.

Löve A. 1969. IOPB Chromosome Number Reports. XXII. Taxon 18: 433-442.

Löve A. 1976. IOPB chromosome number reports LIII. Taxon 25: 483-500.

Löve A. 1977. IOPB chromosome number reports LVI. Taxon 26: 257-274.

Löve A. 1979. IOPB chromosome number reports LXIV. Taxon 28: 391-408.

Löve A. 1980. Chromosome number reports LXVII. Taxon 29: 347-367.

Löve A. 1982. IOPB chromosome number reports LXXIV. Taxon 31: 119-128.

Ma XH, Ma XQ, Li N. 1990. Chromosome observation of some drug plants in Xinjiang. Acta Botanica Boreali Occidentalia Sinica 10: 203-210.

Majovsky J. 1974. Index of chromosome numbers of Slovakian flora, Part 4. Acta Facultatis Rerum Naturalium Universitatis Comenianae Botanica 23: 1-23.

Mangenot S, Mangenot G. 1957. Nombres chromosomiques nouveaux chez divers Dicotylédones et Monocotylédones dAfrique occidentale. Bulletin du Jardin Botanique de l'État 27: 639-654.

Mangenot S, Mangenot G. 1962. Enquête sur les nombres chromosomiques dans une collection d'espèces tropicales. Bulletin de la Société Botanique de France 109: 411-447.

Marhold K. 2006. IAPT/IOPB chromosome data 1. Taxon 55: 443-445.

Mehra PN. 1976. Cytology of Himalayan hardwoods. Sree Saraswaty Press, Calcutta, India.

Morawetz W. 1981. Zur systematischen Stellung der Gattung Prockia: Karyologie und Epidermisskulptur im Vergleich zu Flacourtia (Flacourtiaceae), Grewia (Tiliaceae) und verwandten Gattungen. Plant Systematics and Evolution 139: 57-76.

Neumann A, Polatschek A. 1972. Cytotaxonomischer beitrag zur gattung *Salix*. Annalen des Naturhistorischen Museums in Wien 76: 619-633.

Peto FH. 1938. Cytology of poplar species and natural hybrids. Canadian Journal of Research 16: 445-455.

Petrovsky VV, Zhukova PG. 1983. Chromosome numbers, morphology, ecology and taxonomy of willows of the Northeast of Asia. Botanicheskii Zhurnal 68: 29-38.

Pogan E, Czapik R, Jankun A, Kuta E. 1982. Further studies in chromosome numbers of Polish Angiosperms. Part XV. Acta Biologica Cracoviensia. Series Botanica, 24.

Probatova NS. 1995. Chromosome numbers in some species of vascular plants from the Russian Far East. Botanicheskii Zhurnal 80: 85-88.

Qi LW, Zhang SG, Han SY, Chen CB, Li XL, Song WQ, Chen RY. 2004. Karyotype comparison of *Populus* sect. *Tacamahaca*. Acta Botanica Yunnanica 26: 537-542.

Raven PH, Kyhos DW, Hill AJ. 1965. Chromosome numbers of spermatophytes, mostly Californian. Aliso: A Journal of Systematic and Evolutionary Botany 6: 105-113.

Rice A, Glick L, Abadi S, Einhorn M, Kopelman NM, Salman-Minkov A, Mayzel J, Chay O, Mayrose I. 2015. The Chromosome Counts Database (CCDB) - a community resource of plant chromosome numbers. New Phytologist 206: 19-26.

Rudyka EG. 1990. Chromosome numbers of vascular plants from the various regions of the USSR. Botanicheskii Zhurnal 75: 1783-1786.

Sanders RW, Stuessy TF, Rodriguez R. 1983. Chromosome numbers from the flora of the Juan Fernandez Islands. American journal of botany 70: 799-810.

Singhal VK, Gill BS, Bir SS. 1985. Cytology of woody species. Proceedings: Plant Sciences 94: 607-618.

Smith EC. 1943. A study of cytology and speciation in the genus *Populus* L. Journal of the Arnold Arboretum 24: 275-305.

Suda Y, Argus GW. 1968. Chromosome numbers of some north American *Salix*. Brittonia 20: 191-197.

Suda Y, Argus GW. 1969. Chromosome numbers of some North American arctic and boreal *Salix*. Canadian Journal of Botany 47: 859-862.

Suda Y. 1963. Cytological observations on triploid *Salix gracilistyloides* mstr. Science reports of the Tohoku University 4th Ser. Biology 29.

Sun BY, Park JH, Kwak MJ, Kim CH, Kim KS. 1996. Chromosome counts from the flora of Korea with emphasis on Apiaceae. Journal of Plant Biology 39: 15-22.

Thibault J. 1998. Nuclear DNA amount in pure species and hybrid willows (*Salix*): a flow cytometric investigation. Canadian Journal of Botany 76: 157-165.

Vachova M. 1978. Index of Chromosome numbers of Slovakian flora Part 6. Acta Fac. Rerum Nat. Univ. Comenianae, Bot. 26: 1-18.

Wilkinson J. 1944. The cytology of *Salix* in relation to its taxonomy. Annals of Botany 8: 269-284.

Yurtsev BA. 1982. Chromosome numbers of some plants of the northeastern Yakutia (the Drainage of the Indigerka River in its middle reaches). Botanicheskii Zhurnal 67: 778-787.

Zakirova RO. 1999. Chromosome numbers of some Alliaceae, Salicaceae, Polygonaceae, and Chenopodiaceae of the South Balkhash territory. Citologija 41: 1064.

Zhang CS. 1998. A preliminary study on making plant chromosomal specimens using peppermint oil compound as pretreatment agent. Journal of Wuhan Botanical Research 16: 280-282.

Zhang SG, Chen CB, Han SY, Li XL, Ren JZ, Zhou YQ. 2005. Chromosome numbers of some *Populus* taxa from China. Journal of Systematics and Evolution 43: 539-544.

Zhukova PG. 1967. Chromosome numbers in some species of plants of the north-eastern part of the USSR II. Botanicheskii Zhurnal 52: 982-987.

Zhukova PG. 1973. The chromosome numbers and taxonomy of some plant species from Siberia and far Eat. Botanicheskii Zhurnal 58: 1331-1342.

Zhukova PG. 1980. Chromosome numbers of some Southern Chukotka plant species. Botanicheskii Zhurnal 65: 51-59.
