# Supplementary material for: Frequent ploidy changes in Salicaceae indicates widespread sharing of the salicoid whole genome duplication by the relatives of Populus L. and Salix L
Source: BMC Plant Biol. 2021 Nov 13;21:535. doi: 10.1186/s12870-021-03313-x (PMC8590345; doi:10.1186/s12870-021-03313-x)
Supplement: Supplementary file 2 — Additional file 2. [file 12870_2021_3313_MOESM2_ESM.doc]

Table S2. The 1C nuclear DNA amounts of Salicaceae obtained from Plant DNA C-values database (https://cvalues.science.kew.org/).

| Latin name | DNA amount 1C (pg) | Original reference |
| --- | --- | --- |
| *Casearia bourdillonii* Mukh. | 0.63 | Ohri et al.,2004 |
| *Populus* *alba* L. | 0.52 | Horjales et al.,2003 |
| *P. balsamifera* L. | 0.54 | Bai et al.,2012 |
| *P. canescens* (Aiton) Sm. | 0.47 | Siljak-Yakovlev et al.,2010 |
| *P. deltoides* W. Bartram ex Marshall | 0.49 | Bai et al.,2012 |
| *P. grandidentata* Michx. | 0.55 | Bai et al.,2012 |
| *P. nigra* L. | 0.49 | Pustahija et al.,2013 |
| *P. tremula* L. | 0.45 | Siljak-Yakovlev et al.,2010 |
| *P. tremuloides* Michx. | 0.55 | Bai et al.,2012 |
| *P. trichocarpa* Torr. & A. Gray | 0.49 | Tuskan et al.,2006 |
| *Salix alba* L. | 0.83 | Thibault,1998 |
| *S. amygdaloides* Andersson | 0.36 | Bai et al.,2012 |
| *S. atrocinerea* Brot. | 0.82 | Thibault,1998 |
| *S. babylonica* L. | 0.77 | Horjales et al.,2003 |
| *S. caprea* L. | 0.4 | Mowforth and Grime,1989 |
| *S. cinerea* L. | 0.85 | Thibault,1998 |
| *S. elaeagnos* Scop. | 0.44 | Pustahija et al.,2013 |
| *S. elegantissima* K. Koch | 0.4 | Olszewska and Osiecka,1984 |
| *S. fragilis* L. | 0.86 | Thibault,1998 |
| *S. nigra* Marshall | 0.41 | Bai et al.,2012 |
| *S. purpurea* L. | 0.43 | Pustahija et al.,2013 |
| *S. pyrenaica* Gouan | 0.48 | Thibault,1998 |
| *S. triandra* L. | 0.4 | Thibault,1998 |
| *S. viminalis* L. | 0.41 | Thibault,1998 |
| *S. viminalis* L. | 0.81 | Thibault,1998 |

Bai C, Alverson WS, Follansbee A, Waller DM. 2012. New reports of nuclear DNA content for 407 U.S. plant species. Annals of Botany: 110: 1623-1629.

Horjales M, Redondo N, Blanco A, Rodríguez MA. 2003. Cantidades de DNA nuclear en árboles y arbustos. Nova Acta Cientifica Compostelana (Bioloxía) 13: 23-33.

Mowforth MA. 1985. Variation in nuclear DNA amounts in flowering plants: an ecological analysis. PhD thesis, University of Sheffield, UK.

Ohri D, Bhargava A, Chatterjee A. 2004. Nuclear DNA amounts in 112 species of tropical hardwoods-New estimates. Plant Biology 6: 555-561.

Olszewska MJ, Osiecka R. 1984 Relationship between 2C DNA content, systematic position & level of DNA endoreplication during differentiation of root parenchyma in dicot shrubs & trees-comparison with herbaceous sp. Biochemie und Physiologie der Pflanzen 179: 641-657.

Pustahija F, Brown SC, Bogunic F, Bašic N, Muratovic E, Ollier S, Hidalgo O, Bourge M, Stevanovic V, Sijak-Yakovev S. 2013. Small genomes dominate in plants growing on serpentine soils in West Balkans, an exhaustive study of 8 habitats covering 308 taxa. Plant and soil, 373: 427-453.

Siljak-Yakovlev S, Pustahija F, Šolic EM, Bogunic F, Muratovic E, Bašic N, Catrice O, Brown SC. 2010. Towards a genome size and chromosome number database of Balkan flora: C-values in 343 taxa with novel values for 242. Advanced Science Letters 3: 190-213.

Thibault J. 1998. Nuclear DNA amount in pure species and hybrid willows (*Salix*): a flow cytometric investigation. Canadian Journal of Botany 76: 157-165.

Tuskan GA, DiFazio S, Jansson S, Bohlmann J, Grigoriev I, Hellsten U, Putnam N, Ralph S, Rombauts S, Salamov A, Schein J, Sterck L, Aerts A, Bhalerao RR, Bhalerao RP, Blaudez D, Boerjan W, Brun A, Brunner A, Busov V, Campbell M, Carlson J, Chalot M, Chapman J, Chen GL, Cooper D, Coutinho PM, Couturier J, Covert S, Cronk Q, Cunningham R, Davis J, Degroeve S, Dejardin A, de Pamphilis C, Detter J, Dirks B, Dubchak I, Duplessis S, Ehlting J, Ellis B, Gendler K, Goodstein D, Gribskov M, Grimwood J, Groover A, Gunter L, Hamberger B, Heinze B, Helariutta Y, Henrissat B, Holligan D, Holt R, Huang W, Islam‐Faridi N, Jones S, Jones‐Rhoades M, Jorgensen R, Joshi C, Kangasjarvi J, Karlsson J, Kelleher C, Kirkpatrick R, Kirst M, Kohler A, Kalluri U, Larimer F, Leebens‐Mack J, Leple J‐C, Locascio P, Lou Y, Lucas S, Martin F, Montanini B, Napoli C, Nelson DR, Nelson C, Nieminen K, Nilsson O, Pereda V, Peter G, Philippe R, Pilate G, Poliakov A, Razumovskaya J, Richardson P, Rinaldi C, Ritland K, Rouze P, Ryaboy D, Schmutz J, Schrader J, Segerman B, Shin H, Siddiqui A, Sterky F, Terry A, Tsai C‐J, Uberbacher E, Unneberg P, Vahala J, Wall K, Wessler S, Yang G, Yin T, Douglas C, Marra M, Sandberg G, Van de Peer Y, Rokhsar D. 2006. The genome of black cottonwood, *Populus trichocarpa* (Torr. & Gray). Science 313: 1596–1604.
